# Energy and endoplasmic reticulum stress induction by gold(III) dithiocarbamate and 2-deoxyglucose synergistically trigger cell death in breast cancer

**DOI:** 10.1016/j.jbc.2024.107949

**Published:** 2024-10-30

**Authors:** Owamagbe N. Orobator, R. Tyler Mertens, Oluwatosin A. Obisesan, Samuel G. Awuah

**Affiliations:** 1 Department of Chemistry, University of Kentucky, Lexington, Kentucky, United States; 2 Center for Pharmaceutical Research and Innovation, Department of Pharmaceutical Sciences, College of Pharmacy, University of Kentucky, Lexington, Kentucky, USA; 3 Markey Cancer Center, University of Kentucky, Lexington, Kentucky, USA; 4 Center for Bioelectronics and Nanomedicine, University of Kentucky, Lexington, Kentucky, USA

**Keywords:** mitochondria, endoplasmic reticulum, gold, breast cancer, TNBC, energy

## Abstract

The elusiveness of triple-negative breast cancer from targeted therapy has redirected focus toward exploiting the metabolic shortcomings of these highly metastatic subtypes of breast cancer. Cueing from the metabolic heterogeneity of TNBC and the exposition of the dual dependence of some TNBCs on OXPHOS and glycolysis for ATP, we herein report the efficacy of cotreatment of TNBCs with an OXPHOS inhibitor, 2a and 2DG, a potent glycolysis inhibitor. 2a-2DG cotreatment inhibited TNBC cell proliferation with IC_50_ of ∼5 to 36 times lower than that of 2a alone and over 5000 times lower than IC_50_ of 2DG alone. 2a-2DG cotreatment suppressed mitochondrial ATP production and significantly induced AMPK activation. Mechanistic studies revealed the distinct yet synergistic contributions of 2a and 2DG to the antiproliferative effect of the cotreatment. While 2a induced apoptotic cell death, 2DG sensitized TNBCs to the antiproliferative effects of 2a *via* endoplasmic reticulum stress induction. Strikingly, the combination of 2a-2DG ablated SUM159 tumors in an orthotopic xenograft mouse model. This study highlights the synergistic effect of a gold-based complex with 2DG and the potential benefit of multimetabolic pathways targeting as an effective therapeutic strategy against TNBCs.

Breast cancer is the most common form of cancer and the second leading cause of cancer-associated deaths in females in the United States. In the United States, breast cancer is projected to account for 31% of all newly diagnosed cancer cases and 15% of cancer-related deaths in women in 2023 (1). Triple-negative breast cancer (TNBC), a highly invasive form of breast cancer, is the most aggressive subtype of breast cancer. It accounts for approximately 15 to 20% of all breast cancer cases (2, 3). TNBC is characterized by the absence of estrogen receptors, progesterone receptors, and human epidermal growth factor receptor-2 expressions (2, 4). The lack of hormone receptors results in the unresponsiveness of TNBCs to conventional hormone targeted therapies currently in use for non-TNBCs, and this leads to limited options for TNBC treatment (5). Furthermore, chemotherapy resistance and a high reoccurrence rate present additional impediments in effectively treating TNBCs (6). Due to its highly proliferative nature, TNBCs require constant energy and metabolic intermediates for macromolecule biosynthesis to sustain proliferation. Therefore, interfering with some of these essential energy and metabolic pathways offers an effective strategy for treating TNBCs.

Glycolysis and oxidative phosphorylation are essential pathways for ATP synthesis in cancer cells (7, 8). The role of aerobic glycolysis in cancer was first described by Otto Warburg, who asserted that due to defective mitochondria, cancer cells depend on glycolysis even in the presence of oxygen, a phenomenon commonly called the Warburg effect (9). This proposition was disputed by Sidney Weinhouse, who maintained that mitochondria are intact despite the increased glycolytic rate in some tumors (10). Recent studies have shown that mitochondria are functional and actively participate in the production of ATPs that cancer cells require for proliferation establishing metabolic heterogeneity in tumors (11, 12, 13).

A key hallmark of cancer is metabolic reprogramming. This phenotype is characterized by the ability of tumors to switch between different energy-generating pathways in response to changes in the tumor microenvironment (14, 15). This plasticity ensures tumor adaptability, survival, and proliferation, even in unfavorable conditions (16). Several studies have reported increased mitochondrial oxygen consumption rates in cancer in response to glycolytic inhibitors (17, 18). Conversely, mitochondrial complex 1 inhibitor, metformin, induces oxygen consumption rate reduction while increasing the rate of glycolysis (17, 18). This energy pathway switch in cancer is an essential driver of tumor chemotherapy resistance and metastasis (19). Therefore, developing novel treatment strategies that exploit these metabolic fluidities is essential in advancing the fight against cancer. 2DG is a competitive inhibitor of the glycolytic enzyme, hexokinase, and it has been found to suppress drug resistance, and induce endoplasmic reticulum stress, cell cycle arrest, and apoptosis (5, 20, 21). However, extremely high concentrations of 2DG are needed to halt cancer proliferation (22).

Following the identification of oxidative phosphorylation (OXPHOS) as a therapeutic target in cancer, novel and repurposed OXPHOS inhibitors have been developed and identified, respectively (11, 23). These synthetic efforts led to the development of OXPHOS-modulating anticancer gold compounds (24). Repurposing of the Food and Drug Administration approved neutral gold compound, auranofin, used for the treatment of rheumatoid arthritis as an anticancer agent has stimulated research toward the discovery and development of gold-derived anticancer agents. We and others have made significant contributions to the discovery of novel gold(I) and gold(III) anticancer agents. Additionally, several studies have shown that perturbation of mitochondrial function in tumor cells by gold complexes induce pronounced anticancer effects *in vitro* and *in vivo* (24). The mitochondrial modulating effect of gold agents is facilitated by the ability of gold complexes to form lipophilic cations, which enables them to accumulate within the mitochondria (25). Experimental data have shown the efficacy of some of these gold compounds against breast cancer, especially the highly metastatic and chemotherapy-resistant TNBCs (26, 27). Specifically, our previously reported compound, 2a (a gold(III) dithiocarbamate complex), selectively inhibits tumor growth *in vitro and in vivo*. 2a inhibits mitochondrial complex I and depletes mitochondrial ATP-linked respiration, thus suppressing TNBC progression (25).

Adopting multitarget drug combinations is a practical therapeutic approach in cancer management (28). Polychemotherapy in breast cancer is associated with reduced toxicity, delayed relapse, increased potency at reduced doses, and increased sensitivity of breast cancer to chemotherapy (29, 30). The combinations of the glycolytic inhibitor, 2-deoxyglucose (2DG), and a complex I inhibitor, metformin, have been reported to suppress the proliferation of TNBCs synergistically (18, 31).

Inspired by the antiproliferative and OXPHOS targeting ability of our previously reported compound, 2a (a gold(III) dithiocarbamate complex) (26), we report the synergistic effect of 2a and 2DG against the viability of TNBCs. This combination inhibited TNBC cell lines with a maximal inhibitory concentration (IC_50_) ∼100 folds lower than the IC_50_ of 2a alone and over 5000 times lower than the IC_50_ of 2DG alone. We sought to gain mechanistic insights into the mode of action of 2a-2DG combination and the distinct contributions of 2a and 2DG alone in TNBC cell death. The 2a-2DG combination inhibited the viability of the TNBC cell lines by inducing energy stress, sustained ER stress, and apoptosis. Furthermore, we demonstrated that 2DG-induced ER stress and a combination of 2a and 2DG-escalated ER stress induction.

## Results

### Combining 2a and 2DG inhibits viability and colony formation in TNBC cells

**Figure 1:**
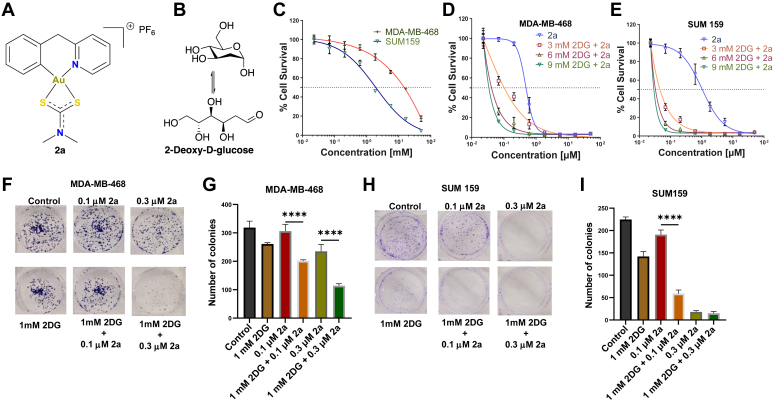
**Effect of 2a and/or 2DG on viability and proliferation in TNBC.** Chemical structures of (*A*) 2a and (*B*) 2DG., (*C*) a dose-response curve showing % cell viability in response to varying concentrations of 2DG in MDA-MB-468 and SUM 159 after 72 h treatment, (*D*) 2a only and 2a-2DG combinations in MDA-MB-468 after 72 h treatment, and (*E*) 2a only and 2a-2DG combinations in SUM 159 after 72 h treatment. Mean ± SD. (n = 6) Cell viability evaluated using MTT assay. Effects of 2a and/or 2DG on colony formation following 2a and/or 2DG treatment on (*F*) and (*G*) (MDA-MB-468, (*H*) and (*I*) SUM 159. Mean ± SD. n = 3. Data presented in *G* and *I* were analyzed by ordinary one-way ANOVA followed by Tukey’s multiple comparison test (∗*p* < 0.05, ∗∗∗*p* < 0.001, ∗∗∗∗*p* < 0.0001). 2DG, 2-deoxyglucose; MTT, (3-(4,5-dimethylthiazol-2-yl)-2,5-diphenyl-2H-tetrazolium bromide); TNBC, triple-negative breast cancer.

**Figure 2:**
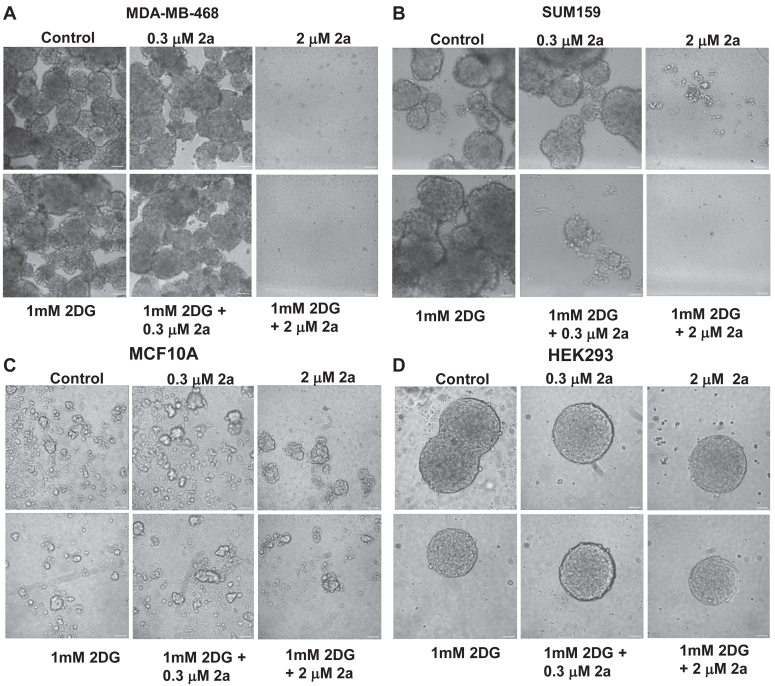
**Effect of 2a and/or 2DG on spheroid formation.** Effects of 2a and/or 2DG on spheroid formation 5 days following 2a and/or 2DG treatment on (*A*) MDA-MB-468 mammospheres, (*B*) SUM159 mammospheres (*C*) MCF10A mammospheres and (*D*) HEK293 spheroids. The scale bar represents 200 μm. 2DG, 2-deoxyglucose.

Increasing evidence supports that metabolic energy plasticity between glycolysis and OXPHOS is crucial for TNBC survival. Considering the mode of action of 2a (Fig. 1*A*) and 2DG (Fig. 1*B*) in TNBC cells, we hypothesized that the combination of 2a and 2DG would be significantly more effective against TNBC cells compared to 2a and 2DG used as monotherapies. Using (3-(4,5-dimethylthiazol-2-yl)-2,5-diphenyl-2H-tetrazolium bromide) MTT assay, the cytotoxic effects of 2a and 2DG in combination and as monotherapies were tested on MDA-MB-468 and SUM159 cells (Fig. 1, *C*–*E*, and Table 1). The IC_50_ of 2DG on MDA-MB-468 and SUM159 cells were 14.6 mM and 1.94 mM, respectively, after 72 h, whereas the IC_50_ of 2a for both cell lines were 0.48 and 1.06 μM, respectively. Interestingly, the combination of 2a and 2DG showed significantly improved cytotoxic effects on both TNBC cells in a concentration-dependent manner. In MDA-MB-468 and SUM 159 cells, the 2a-2DG combination resulted in ∼ 5- to 14- (34–100 nM) and ∼ 23- to 36-fold (29–46 nM) decrease in IC_50_, respectively, compared to just 2a (Fig. 1, *E* and *F*, and Table 1). Using colony formation assay, we further assessed the antiproliferative effects of 2a and/or 2DG on MDA-MB-468 and SUM159 cell lines. Colony formation was inhibited across the 2a and the 2DG treatment groups; however, this inhibitory effect was more significant in the 2a and 2DG combination groups (Fig. 1, *F*–*I*).

**Table 1:** IC_50_ values of 2a and/or 2DG on MDA-MB-468 and SUM159 after 72 h exposure

Compound	IC_50_ values	
	MDA-MB-468	SUM159
2DG (mM)	14.6 ± 2	1.94 ± 0.6
2a (μM)	0.48 ± 0.2	1.06 ± 0.7
3 mM 2DG + 2a (μM)	0.10 ± 0.08	0.046 ± 0.04
6 mM 2DG + 2a (μM)	0.04 ± 0.03	0.033 ± 0.02
9 mM 2DG + 2a (μM)	0.034 ± 0.02	0.029 ± 0.01

2DG stock was prepared in PBS and 2a was freshly prepared in DMSO and used immediately. DMSO concentration was <1%.

### Combination of 2a and 2DG is selectively active against TNBC mammospheres

Mammospheres are three-dimensional cultures of breast cancer cells that relatively mimic the heterogeneity of breast tumor in patients. It has been widely reported that mammospheres are rich in cancer stem cells, drive metastasis, and representative of chemotherapy resistance. We assessed the effect of 2a and 2DG in combination and as monotherapies on TNBC-derived mammospheres cultured on ultra-low adherent plates. In MDA-MB-468 mammospheres, a significant inhibition of the mammospheres was observed in the 2 μM 2a treated group. While 2DG had no inhibitory effect on mammosphere growth, adding 2DG to this concentration of 2a completely inhibited the mammospheres (Fig. 2, *A* and *B*).

While 2DG alone had no inhibitory effect on SUM159 mammospheres, treatment with 2 μM 2a alone significantly inhibited mammosphere formation. We observed a dose-dependent effect of 2a and 2DG cotreatment on the SUM159 mammosphere (Fig. 2*B*). The inhibition of SUM159 spheroids was observed even at a low cotreatment dose of 1 mM 2DG and 0.3 μM 2a. To determine the selectivity of 2a and 2DG for cancer cells over normal noncancerous cells, we evaluated the effect of 2a and 2DG as monotherapies and in combination, in spheroids formed from MCF10A and HEK293, which are normal human breast epithelial cells and normal human embryonic kidney cells, respectively. We found that the different treatments had no cytotoxic effect on the normal spheroids as the structural integrity of the MCF10A and HEK293 spheroids remained intact across all treatment groups (Fig. 2, *C* and *D*), indicative of a promising safety profile of the 2a and 2DG combination.

### Mechanism of cell death in TNBC exposed to 2a and/or 2DG

**Figure 3:**
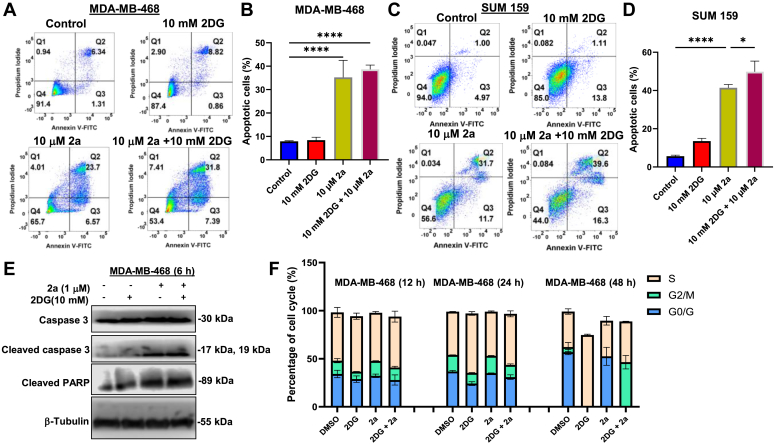
**Mechanism of cell death in 2a and/or 2DG treated TNBC.** Annexin V-PI flow cytometric analysis to assess the effect of 2a (10 μM) and/or 2DG (10 mM) after 18 h treatment on (*A*) and (*C*) MDA-MB-468, (*B* and *D*) SUM 159. n = 3. *E*, Western blot analyses of apoptosis-related proteins in MDA-MB-468 cells treated with 2a (1 μM) and/or 2DG (10 mM) for 6 h. (*F*) Effect of 2a (1 μM) and/or 2DG (5 mM) on cell cycle of MDA-MB-468 cells after 12, 24, and 48 h treatment. Data are plotted as mean ± SD. n = 3. Data presented in *B* and *D* were analyzed by ordinary one-way ANOVA followed by Tukey’s multiple comparison test (∗*p* < 0.05, ∗∗∗*p* < 0.001, ∗∗∗∗*p* < 0.0001). TNBC, triple-negative breast cancer; 2DG, 2-deoxyglucose.

The disruption of the typical structural arrangement of the cell membrane is a molecular event associated with apoptosis. During the process, phosphatidylserine, a lipid with a high affinity for Annexin V, changes orientation from the cytosolic to the exterior part of the membrane. Furthermore, the reduced structural integrity of the damaged membrane facilitates propidium iodide uptake. Thus, using Annexin V-FITC and propidium iodide double staining followed by flow cytometry analysis, we assessed the induction of apoptosis in TNBCs treated with 2DG and/or 2a for 18 h. 2DG (10 mM) induced mild apoptosis in MDA-MB-468 cells, but the percentage of live cells in the 2DG-treated SUM159 cells was significantly reduced compared to the untreated control. 2a (10 μM), both as a monotherapy and in combination with 2DG (10 mM), significantly increased the population of apoptotic cells. However, the proapoptotic potency of 2a was augmented when it was combined with 2DG with values of ∼38% and ∼50% apoptotic cells compared to 2a alone with 35% and 41% for MDA-MB-468 and SUM159, respectively (Fig. 3, *A*–*D*).

To further determine the effect of 2a and/or 2DG on apoptosis, we assessed the expression levels of apoptosis marker proteins, cleaved PARP, and cleaved caspase 3 in MDA-MB-468 cells. Our result shows that 2a (1 μM) alone and in combination with 2DG (10 mM) significantly increased the expression of cleaved PARP and cleaved caspase 3 (Fig. 3*E*) after 6 h; however, there was no significant difference in the protein expression between these two groups. Treatment with 2DG (10 mM) alone did not markedly increase cleaved PARP and cleaved caspase 3.

We determined the effect of 2a (1 μM) and/or 2DG (5 mM) on cell cycle in MDA-MB-468 cells. 2DG significantly increased the population of cells in the S phase compared to the untreated control by 15%, 37.5%, and 102% after treatment for 12 h, 24 h, and 48 h, respectively. After 12 h and 24 h treatment with 2a, we observed an 11% and 4% increase in the G2/M phase, respectively, compared to the control. Interestingly, a combination of 2a and 2DG induced a 700% increase in cell population in the G2 phase and a 24% increase in the S phase after 48 h treatment (Fig. 3*F*). Taken together, a combination of 2a and 2DG induced S phase and G2 phase arrest.

### The combination of 2a and 2DG produces a synergistic effect in TNBC

We decided to investigate whether the effect of the 2a and 2DG combination is synergistic, additive, or antagonistic using the Chou-Talalay method for drug combination (32). This method is based on the law of mass action and provides essential information such as combination index (CI) and dose reduction index (DRI). The CI values describe the interaction between the two compounds. CI < 1, CI = 1, and CI > 1 represent synergistic, additive, and antagonistic effects, respectively. Using the non-constant ratio approach, varying concentrations of 2a were combined with different fixed concentrations of 2DG for MDA-MB-468 and SUM159 (Table 2). Both TNBC cells were treated with selected concentrations of 2DG and the gold complex (2a) for 72 h.

**Table 2:** Combinations of 2a and 2DG used in the synergistic study in MDA-MB-468 and SUM159 cells

2a Concentration	3 mM 2DG	6 mM 2DG	9 mM 2DG
50 μM 2a	50, 3	50, 6	50, 9
16.7 μM 2a	16.7, 3	16.7, 6	16.7, 9
5.56 μM 2a	5.56, 3	5.56, 6	5.56, 9
1.85 μM 2a	1.85, 3	1.85, 6	1.85, 9
0.62 μM 2a	0.62, 3	0.62, 6	0.62, 9
0.21 μM 2a	0.21, 3	0.21, 6	0.21, 9
0.069 μM 2a	0.069, 3	0.069, 6	0.069, 9

**Figure 4:**
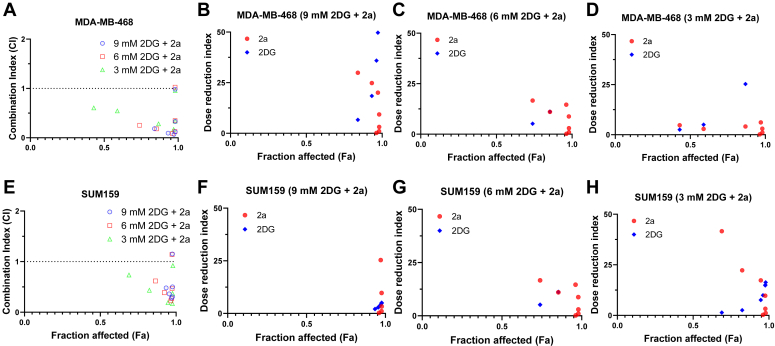
**Effects of combined treatment of TNBC cells with 2a and 2DG.** The Fa-CI plots demonstrate the synergism of 2a and 2DG combinations in (*A*) MDA-MB-468 and (*E*) SUM159, calculated with the Compusyn software. Fa-DRI plots show the reducibility of the 2a and 2DG doses in the combination treatment against MDA-MB-468 (*B*–*D*) and SUM159 (*F*–*H*). Data points represent mean values. 2DG, 2-deoxyglucose; DRI, dose reduction index; TNBC, triple-negative breast cancer.

A broad range of combinations of 2a and 2DG were found to be synergistic in both MDA-MB-468 and SUM159 cells. Out of the 21 different combinations of 2a and 2DG against the MDA-MB-468 cells, 17 combinations exhibited synergistic effects with CI < 1 (Fig. 4*A*). Similar synergistic effects were observed for combinations against SUM159 cells, with 16 out of 21 combinations showing synergism (Fig. 4*E*). A key determinant of the effectiveness of a synergistic combination against cancer is the Fa (fractional inhibition or fraction affected) value. The Fa value represents the percent proliferation inhibition; at 100% inhibition, fa = 1, and 0% inhibition, fa = 0 (33, 34) Synergism is more effective in cancer treatment at high fa values. Interestingly, most of our combinations exhibited synergistic effects even at fa > 0.9 in MDA-MB-468 and SUM159 cells (Fig. 4, *A* and *E*).

DRI describes the number of folds dose-reduction is allowed for individual drugs in a synergistic combination while maintaining efficacy (35, 36). It indicates the safety and potency thresholds of each drug in the combination. For MDA-MB-468 cells, 17 out of 21 total combinations, 2a showed a DRI > 1 for 17 compound combinations, ranging from 1.03 to 29.9 (Figs. 4, *B*–*D* and Tables S1–S3). This indicates that the effective therapeutic doses of 2a in the appropriate combination can be reduced by ∼ 1 to ∼30 times. A similar trend was observed for the SUM159 cells, with 2a having a DRI > 1 in 18 out of 21 combinations. (Fig 4, *F*–*H* and Tables S4–S6). This signifies that 2a in the combination can be reduced to avoid toxicity while maintaining potency.

### The combination of 2a and 2DG induces metabolic energy stress in TNBC

**Figure 5:**
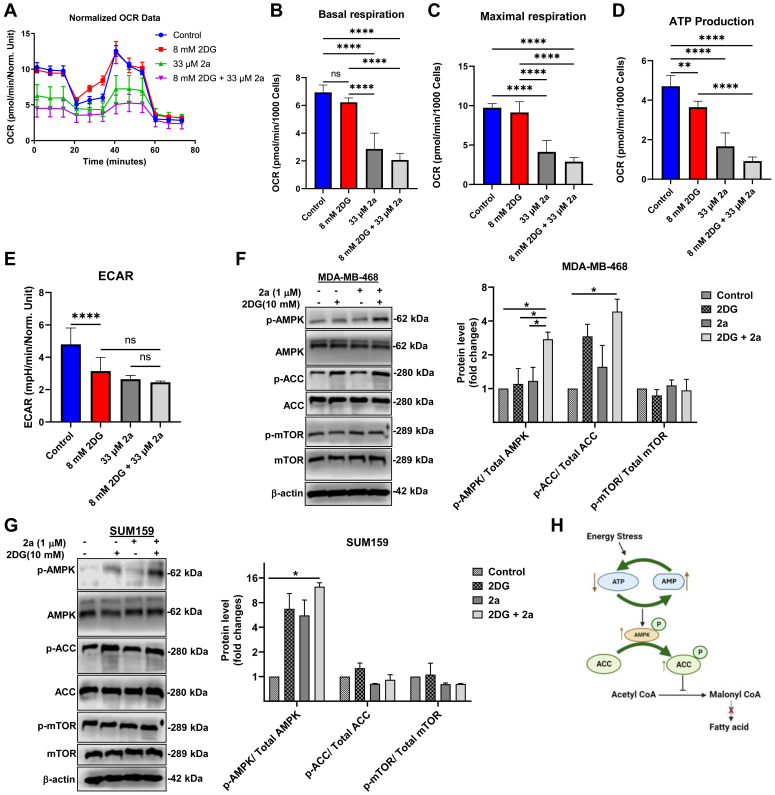
**Combination of 2a and 2DG inhibits oxygen consumption and mitochondria-ATP production.** (*A*) OCR data following treatment of MDA-MB-468 cells with 2a and/or 2DG for 24 h. Extrapolated parameters from mito stress test upon treatment of MDA-MB-468 cells with 2a and/or 2DG showing (*B*) basal respiration, (*C*) maximal respiration, (*D*) ATP production, and (*E*) rate of glycolysis. Western blot showing key energy stress marker, AMPK, and proteins associated with AMPK pathway following treatment with 2a and/or 2DG for 6 h in (*F*) MDA-MB-468 and (*G*) SUM159. *H*, mechanism of AMPK and ACC phosphorylation. Data are plotted as the mean ± SD. (n = 8). Data presented in *B*–*G* were analyzed by ordinary one-way ANOVA followed by Tukey’s multiple comparison test (∗*p* < 0.05, ∗∗*p* < 0.01, ∗∗∗*p* < 0.001, ∗∗∗∗*p* < 0.0001. n.s. = not significant). 2DG, 2-deoxyglucose; ACC, acetyl CoA carboxylase; AMPK, AMP-activated protein kinase; OCR, oxygen consumption rate.

Given our knowledge of the metabolic heterogeneity of TNBCs and the exposition by us and others on the dual dependence of some cancer cells on both OXPHOS and glycolytic energy sources for proliferation and survival (13, 37), we decided to assess the effect of 2a and 2DG on the bioenergetic profile of MDA-MB-468 cells using mito stress assay. This assay involves subjecting MDA-MB-468 cells to 2a and/or 2DG treatment for a defined period, followed by injecting known electron transport chain inhibitors (Fig. 5*A*) and measuring key parameters. As expected, 2a significantly inhibited basal respiration, maximal respiration, and ATP synthesis (Fig. 5, *B*–*D*), indicative of perturbed mitochondrial respiration. Interestingly, the rate of glycolysis, as shown by the extracellular acidification rate level, was markedly reduced following treatment with 2a (Fig. 5*E*).

2DG induced a significant reduction in glycolysis, however, there was no significant change in basal and maximal respiration compared to the control. Treatment of MDA-MB-468 cells with a combination of 2a and 2DG reduced ATP production, basal and maximal respiration, compared to 2a and 2DG alone.

The role of AMP-activated protein kinase (AMPK) in response to energy stress has been thoroughly investigated (38, 39). AMPK is phosphorylated and activated in a state of high AMP: ATP or high ADP: ATP ratio (40, 41). Following its activation, it modulates a cascade of metabolic pathways by inhibiting ATP-consuming processes. AMPK activation facilitates the inhibition of mammalian target of rapamycin (mTOR) and acetyl CoA carboxylase (ACC) (Fig. 5*H*), two important proteins that promote cell proliferation and fatty acid synthesis, respectively (42, 43). To gain more insight into the role of 2a and/or 2DG in inducing energy stress in TNBCs, we assessed the expression levels of P-AMPK, P-mTOR, and P-ACC using immunoblotting analysis (Fig. 5, *E*–*G*). The result showed mild phosphorylation of AMPK in the 2a only and 2DG-only treated groups in MDA-MB-468 and SUM159 cells. However, the combination of 2a and 2DG significantly increased the activation of AMPK after 6 h treatment in both cell lines. In the 2a-2DG combination treated MDA-MB-468 cells, ACC was significantly phosphorylated and inhibited following AMPK activation. These effects were not observed in the SUM159 cells. The results suggest that the 2a-2DG combination induced energy stress in TNBCs. However, the AMPK activation in SUM 159 did not significantly induce phosphorylation of ACC.

### 2a and 2DG combination treatment induces endoplasmic reticulum stress in TNBC

**Figure 6:**
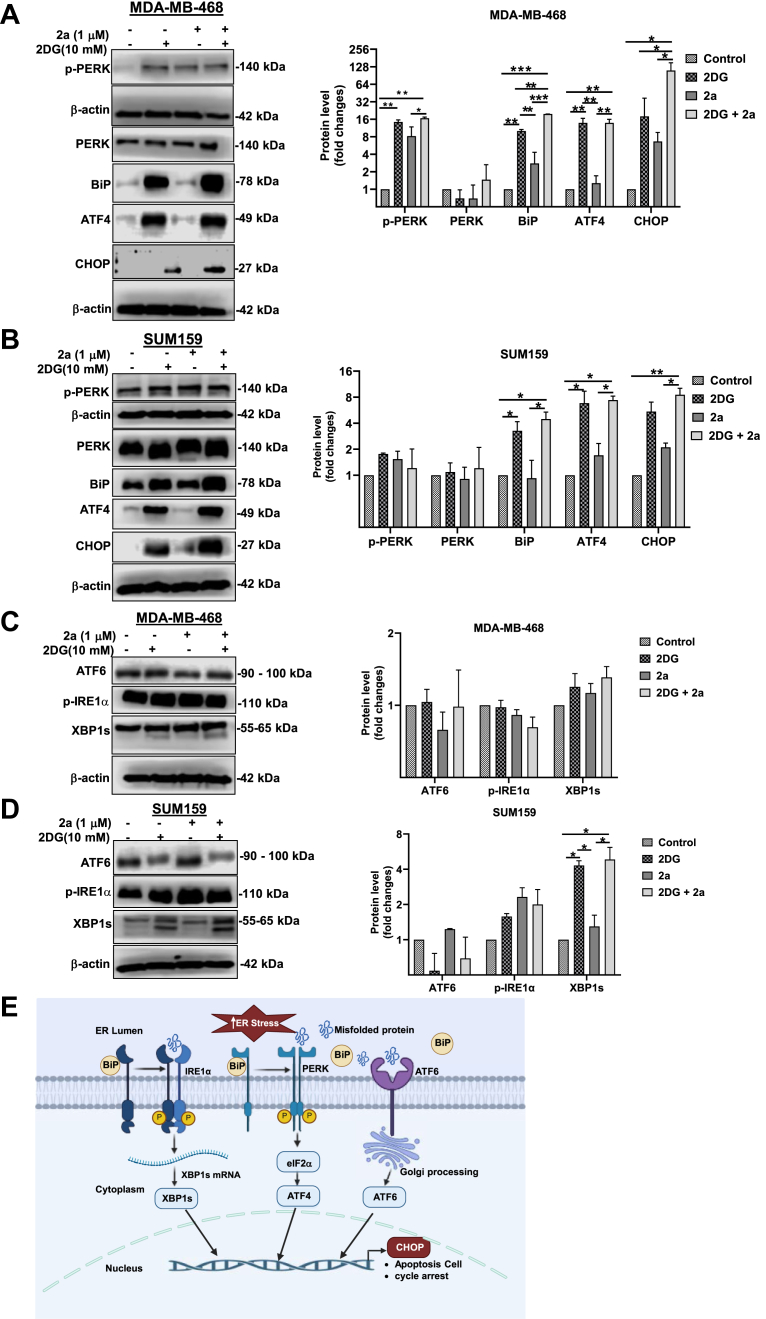
**Cotreatment of 2a and 2DG intensifies induction of ER stress in TNBC.** Western blot showing the expression of phosphorylated protein kinase R-like ER Kinase (p-PERK), protein kinase R-like ER kinase (PERK), binding immunoglobulin protein (BiP), activating transcription factor 4 (ATF4), C/EBP homologous protein (CHOP), activating transcription factor 6 (ATF6), phosphorylated inositol-requiring enzyme 1 alpha (p-IRE1α), and x-box binding protein 1, spliced form (XBP1s) in (*A*) and (*C*) MDA-MB-468 cells and (*B*) and (*D*) SUM159 after 6 h treatment with 2a and/or 2DG (*E*) mechanism of ER stress activation through the three arms of the pathway. Data presented in *A*–*D* were analyzed by ordinary one-way ANOVA followed by Tukey’s multiple comparison test (∗*p* < 0.05, ∗∗*p* < 0.01, ∗∗∗*p* < 0.001). 2DG, 2-deoxyglucose; TNBC, triple-negative breast cancer.

The ER has gained traction as a potential therapeutic target in TNBC in recent years (44, 45, 46). As an organelle, ER carries out functions such as storing calcium, regulating lipid metabolism, protein synthesis, and folding *via* N-linked glycosylation by GDP-mannose. Due to its role, the ER can be subjected to stress caused by misfolded proteins. The metabolism of 2DG results in the accumulation of GDP-2DG, a potent competitor of GDP-mannose. Therefore, GDP-2DG interferes with N-linked glycosylation, leading to protein misfolding (47, 48). The protein folding function of the ER is an active process, usually requiring ATP (49, 50). Steady ER ATP supply is essential for the chaperone proteins involved in protein folding. It has been shown that inhibiting mitochondrial ATP production could induce some levels of ER stress (51). Cueing from the role of 2DG in protein misfolding and the reported importance of mitochondrial ATP in the proper protein folding, we hypothesized that the combination of 2DG and 2a will intensify the ER stress induction in TNBC cells compared to 2a and 2DG used as monotherapies. To test this hypothesis, we decided to assess the effect of 2DG in ER stress and if the combination of 2a with 2DG could increase the ER stress induction in TNBCs. We also gleaned insight into the preferred pathways of ER stress induction following treatment with 2a and/or 2DG. We carried out immunoblotting for key proteins involved in the three ER stress pathways: the PERK/ATF4/CHOP pathway, the ATF6 pathway, and the IRE1α/XBP1 pathway. 2DG induced strong expression of binding immunoglobulin protein (BiP), an indicator of ER stress, in both MDA-MB-468 and SUM159 cells. However, BiP was only mildly expressed in the 2a alone treated group (Fig. 6, *A* and *B*) in both cell lines, while the combination of 2a with 2DG significantly increased the expression of BiP compared to other treatment groups in the MDA-MB-468 cells.

The expression of BiP during ER stress in response to misfolded protein results in protein kinase R-like ER kinase (PERK) activation, followed by a cascade of activation events such as activating transcription factor 4 (ATF4) and C/EBP homologous protein (CHOP) activation (6E). In MDA-MB-468 cells, treatment with 2DG alone resulted in the phosphorylation of PERK to a markedly higher level than that of control but not different from the 2a treatment group. However, combining 2a and 2DG induced a significantly higher phosphorylated PERK (p-PERK) expression than the control and 2a groups. In SUM159, there was no significant difference in phosphorylated PERK expression across all treatment groups. This underscores the cell-line dependent response of the ER to these treatments. For both MDA-MB-468 and SUM159, 2DG alone and in combination with 2a induced ATF4 activation (Fig. 6, *A* and *B*). CHOP is expressed following prolonged or intense ER stress, and its expression increases the sensitivity of TNBC to anticancer drugs, induces cell cycle arrest, and the susceptibility of cancer cells to apoptosis (52). We observed that the cotreatment of MDA-MB-468 and SUM159 cells with 2a and 2DG significantly intensified CHOP expression compared to other treatment groups. To understand the effect of 2a and 2DG alone and in combination on ATF6-mediated ER stress, we immunoblotted for ATF6 in MDA-MB-468 and SUM159 cells. We observed that there was no significant difference in the expression level of ATF6 across all treatment groups in both cell lines (Fig. 6, *C* and *D*). This suggests that the ATF6 pathway is not involved in the induction of ER stress following treatment with 2a and 2DG. Studies have reported the activation of p-IRE1α in response to BiP and the subsequent expression of spliced form of x-box binding protein 1 (XBP1s) (Fig. 6*E*) during ER stress (53, 54). We sought to determine if the IRE1α/XBP1 pathway mediates ER stress induction following treatment with 2a and/or 2DG. We observed that there was no significant expression of p-IRE1α and XBP1s in the treated groups compared to the control in MDA-MB-468. In SUM159 cells, though there was no marked p-IRE1α expression, however, there was an increased expression of XBP1s in the 2DG and 2a -2DG treatment groups. Furthermore, we observed that this increase in XBP1s expression in SUM159 can be attributed to 2DG because the 2a -2DG combination conferred no expression advantage. There exists some crosstalk between XBP1s and ATF4 in some cells, as XBP1s transcriptionally upregulate ATF4. Inhibition of XBP1s and conditional knockdown of XBP1s have been reported to decrease ATF4 expression markedly, while the activation of XBP1s resulted in ATF4 expression in mouse bone marrow mesenchymal stem cells (55). This XBP1s – ATF4 interaction could be contributing to the ATF4 expression observed in the 2DG and 2a -2DG treatment groups of SUM159 cells. Taken together, 2DG induces ER stress, and cotreatment with 2a potentiates ER stress induction in TNBC in cell-line dependent ER stress pathway.

### 2a and 2DG combination is effective against TNBC progression *in vivo*

**Figure 7:**
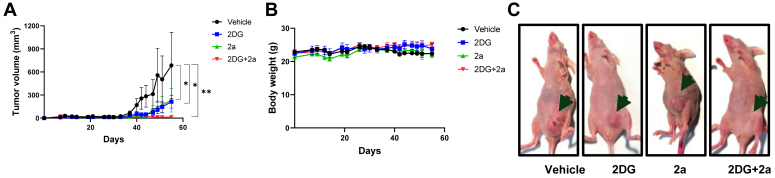
**Combination of 2a and 2DG inhibits TNBC progression *in vivo*.***A*, change in SUM159 tumor volume following 2a and/or 2DG administration for 27 days. *B*, body weight of mice following 2a and/or 2DG administration for 27 days. *C*, representative images of the tumor across all the mice groups. Data are plotted as mean ± SEM. n = 3. Data were analyzed by two-sided Student’s *t* test (∗*p* < 0.05, ∗∗*p* < 0.01). 2DG, 2-deoxyglucose; TNBC, triple-negative breast cancer.

Having shown the effectiveness of the 2a and 2DG combination against TNBC mammospheres (Fig. 2, *A* and *B*), we then sought to investigate the efficacy of this combination *in vivo* using the TNBC xenograft model. SUM159 orthotopic xenograft tumors were established in the fourth mammary fat pad of athymic nude mice. The mice were administered 2a alone (5 mg kg^−1^) or 2DG alone (500 mg kg^−1^) or 2a - 2DG combination or vehicle, intraperitoneally. The analysis of the tumor growth indicated 66%, 69%, and 97% tumor growth inhibition in the 2DG alone, 2a alone, and 2a - 2DG combination treatment groups, respectively (Fig. 7, *A* and *C*).

Furthermore, whereas no marked loss of body weight was observed across the treatment groups, an increase was observed in the 2a-2DG combination treatment group compared to the control group (Fig. 7*B*).

## Discussion

The unresponsiveness of TNBCs to targeted therapy remains a significant impediment to its management. In attempts to address this challenge, there have been concerted efforts toward developing novel antiproliferative compounds and therapeutic approaches to effectively treat these highly metastatic and invasive subtypes of breast cancer. Leveraging on the new insight into the distinct metabolic profile and heterogeneity of TNBCs, there has been an increase in treatment strategies targeted at perturbing metabolism in TNBCs. These include developing leads that inhibit the OXPHOS pathways and adopting multipathway targeting. New synthetic approaches have led to the development of gold complexes that perturb the mitochondria and modulate the OXPHOS pathway. These complexes inhibit respiration and OXPHOS-linked ATP production in TNBCs (24, 26). Dual inhibition of the glycolytic and OXPHOS pathways poses an effective tactic in TNBC treatment due to the significant contribution of both pathways to cellular energy required for proliferation in TNBCs.

Herein, we report our findings on the cotreatment of TNBCs with 2a and 2DG, which we found to be significantly more potent against MDA-MB-468 and SUM159 cells than 2a and 2DG used as monotherapies. This compound combination significantly inhibited proliferation and colony formation in the two cell lines. We rationalized that the individual compounds in the cotreatment would synergistically contribute to TNBCs cell death *via* distinct mechanisms. We observed that the 2a component of the combination was the main driver of apoptosis, although this apoptotic effect was potentiated when 2DG was introduced.

Studies have shown the therapeutic benefit of combining 2DG with other metabolic compounds against cancer bioenergetics. 2DG has been reported to synergize with 5000 μM of metformin to suppress mitochondrial respiration and ATP synthesis (18). We observed that the cotreatment of MDA-MB-468 cells with 2DG and 33 μM 2a reduced respiration and ATP production compared to the individual compounds used alone. As expected, 2a-2DG combination treatment resulted in significant AMPK phosphorylation. Phosphorylation of AMPK represents a reliable signal for ATP depletion (40, 43). In consonance with other studies that phospho-AMPK overexpression phosphorylated ACC (43), a critical protein for fatty acid synthesis, cotreatment with 2a and 2DG depleted p-mTOR and inhibited ACC in SUM159 and MDA-MB-468 cells, respectively. Considering the indispensable role of ATP in cell survival, selectively inhibiting ATP production in cancer cells over normal cells is crucial. The high proliferative nature of TNBC cells increases their demand for ATP and sensitivity to ATP scarcity compared to normal cells (56). Energy-targeting anticancer combination therapies leverage this energy gluttony to selectively target cancer cells over normal cells. Furthermore, the lower energy demand in noncancerous cells ensures their survival during ATP scarcity and facilitates the switch of these cells to alternative low ATP turnover pathways.

To deepen our understanding of the anticancer contribution of 2DG in the codrug treatment, we assessed the effect of 2a and/or 2DG on ER stress. ER stress induction has been reported to sensitize tumor cells to anticancer drugs, facilitate apoptosis induction, and induce cell cycle arrest (57, 58, 59, 60). 2DG treatment of MDA-MB-468 and SUM159 resulted in the expression of ER stress markers, such as BiP, ATF4, and CHOP, but treatment with 2a alone did not induce the expression of these markers. However, we observed that cotreatment with 2a and 2DG induced an amplified expression of these markers compared to just 2DG alone. This suggests that 2DG may be contributing to the antiproliferative effect of the combination *via* ER stress induction; however, the ER stress is amplified in the presence of 2a.

TNBC orthotopic xenografts of human origin represent translational models to test the efficacy of anticancer agents because these tumors are inoculated in their native sites, the mammary fat pad. The combination of 2a and 2DG exhibited immense anticancer and tolerability potentials against the TNBC xenografts. Besides efficacy, a crucial therapeutic benefit of combination therapy is reduced toxicity due to the dose reduction of one or more agents. Previous *in vivo* studies with 2a in treating a breast cancer mouse model at 10 mg kg^−1^ dose revealed accumulation of the compound mainly in the liver and kidney, which are the central clearance and excretory organs, and histological assessment of the liver, kidney, spleen, lungs, and heart tissues of the control and 2a treated groups showed no marked difference, indicating the relative safety of the compound (25). Incorporating 2DG in this current study allowed for a dose reduction of the 2a (5 mg kg^−1^) in the *in vivo* study, thus improving the tolerability of the treatment. We posit that the combined targeting of ER stress and metabolic pathways holds promise for effective therapeutic management of aggressive cancer such as TNBC.

## Experimental procedures

### Cell culture

MDA-MB-468, SUM159, MCF10A, and HEK293 cell lines used for this study were purchased from the American Type Culture Collection (ATCC) and grown in a humidified incubator at 37 °C with 5% CO_2_. MDA-MB-468 and HEK293 cells were maintained in Dulbecco’s modified Eagle’s medium supplemented with 10% fetal bovine serum, 1% penicillin/streptomycin, and 1% amphotericin. SUM159 cells were maintained in Dulbecco’s modified Eagle’s medium supplemented with 10% fetal bovine serum, 1% penicillin/streptomycin, 1% amphotericin, and 5 μg/ml insulin. MCF10A cells were maintained in Lonza’s mammary epithelial cell growth medium supplemented with Lonza’s mammary epithelial cell growth medium bullet kit containing, bovine pituitary extract, human epidermal growth factor, insulin, hydrocortisone and Gentamicin-Amphotericin (GA-1000). All the supplements, trypsin-EDTA, and PBS used for this study were purchased from Corning Inc and used as purchased.

### Cell viability assay

MDA-MB-468 and SUM159 cells were seeded at a density of 2000 cells/ml in 100 μl in 96 well plates. The cells were incubated at 37 °C and allowed to adhere overnight. 2a was prepared as a 500 μM stock using dimethylsulfoxide (DMSO) and media and diluted to 200 μΜ working concentration. Threefold serial dilutions were made to a total of seven concentrations. 2DG was prepared as a 300 mM stock in PBS and Media and diluted to a working concentration of 200 mM. For IC_50_ of the two compounds, 2a and 2DG were diluted to the highest concentrations of 150 μM and 150 mM, respectively, and then three-fold dilutions were made to a total of seven concentrations for each compound. The compounds were added such that the highest concentrations of 2a and 2DG in the wells were 50 μM and 50 mM, respectively. For 2a and 2DG cotreatment, 3 mM, 6 mM, and 9 mM 2DG were separately cotreated with 2a (highest concentration of 50 μM diluted 3-folds to a total of seven concentrations). The treated plates were incubated at 37 °C for 72 h. After incubation, 100 μl of MTT was added at a final concentration of 0.5 mg/ml and incubated for 4 h. After 4 h, the MTT solution was pipetted out of the plates and replaced with 100 μl DMSO. Then, absorbance was measured at a wavelength of 570 nm. Data were analyzed and plotted with GraphPad Prism software (https://www.graphpad.com/).

### Spheroid formation assay

MDA-MB-468, SUM159, MCF10A, and HEK293 cells were trypsinized, pelleted, and resuspended as single cell suspension in MammoCult human medium (STEMCELL Technologies) supplemented with heparin (4 μg/ml, STEMCELL Technologies) and hydrocortisone (0.48 μg/ml, STEMCELL Technologies). Cells were plated at a density of 4000 cells/ml per well in 24-well ultralow attachment plates. Mammospheres were allowed to form for 48 h. The spheroids were treated as control, 2a, 2DG, and 2a + 2DG for 5 days. The spheroids were photographed using the Olympus IX70 inverted fluorescence microscope.

### Apoptosis analysis

MDA-MB-468 and SUM159 cells were seeded at a density of 5 × 10^5^ cells/ml in 6-well plates. The cells were incubated at 37 °C and allowed to adhere overnight. 2a was prepared as a stock of 5 mM in DMSO. 2DG was prepared as 100 mM stock in PBS. 10 μM 2a, 10 mM 2DG, and a combination of 10 μM 2a + 10 mM 2DG were added to the respective wells and incubated for 18 h. After incubation, media were collected in 15 ml centrifuge tubes. The plates were rinsed with 2 ml PBS and collected in the 15 ml tubes. The cells were detached from the plates by trypsinization and added to the appropriate 15 ml tubes. The cells were pelleted by centrifuging for 5 min. The supernatants were discarded, and the cells were resuspended in 5 ml of PBS and pelleted by centrifugation. The supernatant was discarded, and the cells were suspended in 0.3 ml of Annexin binding buffer. To each tube, 5 μl of Annexin V-FITC and 5 μl propidium iodide (PI) were added and incubated in the dark for 5 min before flow cytometry analysis.

### Immunoblotting

MDA-MB-468 and SUM159 cells were seeded at a density of 5 × 10^5^ cells/ml in 6-well plates. The cells were incubated at 37 °C and allowed to adhere overnight. The cells in the appropriate wells were treated with 1μΜ 2a, 10 mM 2DG, and a combination of 1 μM 2a + 10 mM 2DG and incubated for 6 h. After incubation, the cells were washed twice with PBS, and Laemmli buffer was added. The cells were collected by scrapping into a 1.5 ml centrifuge tube. The lysates were incubated at 90 °C for 5 min on a heating block. The protein mix was separated by 4 to 20% SDS-polyacrylamide gel electrophoresis (35 min, 200V). After separation, the proteins were transferred to polyvinylidene fluoride membranes (1 h, 100V), followed by membrane blocking using 5% (w/v) bovine serum albumin in tris-buffer saline with tween-20 for 1 h at room temperature. This was followed by incubation with the appropriate primary antibody overnight at 4 °C. The following day, the membranes were washed thrice with tris-buffer saline with tween-20 for 5 min and then incubated with horseradish peroxidase-conjugated secondary antibodies (at room temperature for 1 h) prepared in 5% bovine serum albumin blocking solution. The membranes were placed in Pierce-enhanced chemiluminescence substrate and visualized with a Bio-Rad imager. Antibodies used for this study were purchased from Cell Signaling Technology, Proteintech, and Novus Biologicals.

### Cell cycle

MDA-MB-468 and SUM159 cells were seeded at a density of 2.5 × 10^5^ cells/ml in 6-well plates. The cells were incubated at 37 °C and allowed to adhere overnight. The cells in the appropriate wells were treated with 1μΜ 2a, 5 mM 2DG, and a combination of 1 μM 2a + 5 mM 2DG and incubated for 12, 24, and 48 h. After incubation at the different time points, media were collected in 15 ml centrifuge tubes. The plates were rinsed with 2 ml PBS and collected in the 15 ml tubes. The cells were detached from the plate by trypsinization, neutralized by media, and added to the appropriate 15 ml tubes. The cells were pelleted by centrifuging for 5 min. The supernatants were discarded, and the cells were resuspended in 1 ml of PBS, then transferred to a 1.5 ml Eppendorf tube and centrifuged at 2000 rpm for 5 min to form pellets. The pellets were suspended in 70% ethanol in PBS solution, and the solution was stored at 4 °C until ready for analysis. After collecting all treatment, the cells were centrifuged at 2000 rpm for 5 min to form pellets. The cells were washed twice with PBS (1 ml) and suspended in 200 μl of a 50 mg/ml PI solution, and 50 μl of RNase solution (100 mg/ml) was added. The solutions were then filtered through a 5 ml polystyrene round bottom fluorescence-activated cell sorting tube with a cell-strainer cap. The samples were analyzed with fluorescence-activated cell sorting. The experiment was conducted in triplicates, with percentages plotted as the mean ± SD (n = 3).

### Colony formation assay

MDA-MB-468 and SUM159 cells were plated in 6-well plate at 1000 cells per well. Cells were cultured with the appropriate concentration of 2a alone, 2DG alone and a combination of 2a and 2DG for 10 days. After incubation, the cells were fixed with 4% paraformaldehyde for 1 h and stained with crystal violet for 20 min, and the number of colonies was counted using ImageJ software (https://imagej.net/).

### Bioenergetics measurement with Seahorse XF96 analysis

MDA-MB-468 cells were seeded at 20,000 cells/well for the Seahorse XF96 experiments. The cells were seeded a day before the experiment in a 100 μl volume per well and incubated overnight at 37 °C. The cells in the appropriate wells were treated with 33 μΜ 2a, 8 mM 2DG, and a combination of 33 μM 2a + 8 mM 2DG and incubated for 24 h. This was followed by injection of oligomycin (1.5 μM), carbonyl cynanide-4-(trifluoromethoxy)phenylhydrazone (0.6 μM) and rotenone/antimycin A (0.5 μM). The metabolic parameters were calculated based on readings from a minimum of three wells.

### Synergy analysis

Synergistic studies were carried out on different combinations of 2a and 2DG using the nonconstant ratios approach described by the Chou-Talalay method (32). MDA-MB-468 and SUM159 cells were seeded at a density of 2000 cells/ml in 100 μl in 96 well plates. The cells were incubated at 37 °C and allowed to adhere overnight. 2a was prepared as a 500 μM stock using DMSO and media and diluted to 200 μΜ working concentration. Threefold serial dilutions were made to a total of seven concentrations. 2DG was prepared as a 300 mM stock in PBS and Media and diluted to a working concentration of 200 mM. For IC_50_ of 2a alone and 2DG alone, 2a and 2DG were diluted to the highest concentrations of 150 μM and 150 mM, respectively, and then three-fold dilutions were made to a total of seven concentrations for each compound. The compounds were added such that the highest concentrations of 2a and 2DG in the wells were 50 μM and 50 mM, respectively. For 2a and 2DG cotreatment, 3 mM, 6 mM, and 9 mM 2DG were separately cotreated with 2a (highest concentration of 50 μM diluted to a total of seven concentrations). The treated plates were incubated at 37 °C for 72 h. After incubation, 100 μl of MTT was added at a final concentration of 0.5 mg/ml and incubated for 4 h. After 4 h, the MTT solution was pipetted out of the plates and replaced with 100 μl DMSO. Then, absorbance was measured at a wavelength of 570 nm. The CompuSyn software (https://compusyn.software.informer.com/1.0/) was used to determine the CI and DRI. Synergistic, additive, and antagonistic effects were represented by CI < 1, CI = 1 and CI > 1, respectively.

### *In vivo* experiments

Six-week-old female athymic nude mice were purchased from Jackson Laboratory and allowed to acclimatize for 1 week before inoculation. Following acclimatization, 2,000,000 SUM159 cells were mixed with a 1:1 ratio of Matrigel and PBS, and then transplanted into the fourth mammary fat pad of the athymic nude mice. Once the tumors were established, mice were treated with 500 mg kg^−1^ 2DG (formulated in PBS), 5 mg kg^−1^ 2a (formulated as 1% DMSO, 9% kolliphor, and 90% deionized water), or a combination of 2DG and 2a or vehicle (1% DMSO, 9% kolliphor, 90% deionized water) respectively *via* intraperitoneal injection two times per week. Body weight was recorded. Tumor volume was measured using a caliper and calculated using the formula: tumor volume = length X width ^2^/2. All mice were maintained in a pathogen-free environment under the care of DLAR of the University of Kentucky. Our study was performed in compliance with the NIH guidelines (NIH Publication No. 85–23 Rev. 1985) for the care and use of laboratory animals, and all experimental procedures were monitored and approved by the Institutional Animal Care and Use Committee (IACUC) of the University of Kentucky (USA).

## Data availability

All data described is contained within the manuscript. Raw data will be made available upon request to the corresponding author.

## Supporting information

This article contains supporting information.

## Conflict of interest

The authors declare the following financial interests/personal relationships which may be considered as potential competing interests: Samuel G. Awuah has patents pending to University of Kentucky Research Foundation.
