# Supplementary material for: Energy and endoplasmic reticulum stress induction by gold(III) dithiocarbamate and 2-deoxyglucose synergistically trigger cell death in breast cancer
Source: J Biol Chem. 2024 Oct 30;300(12):107949. doi: 10.1016/j.jbc.2024.107949 (PMC11647619; doi:10.1016/j.jbc.2024.107949)

**Energy and endoplasmic reticulum stress induction by gold(III) dithiocarbamate and 2-deoxyglucose synergistically trigger cell death in breast cancer**

Owamagbe N. Orobator ^a^, R. Tyler Mertens ^a^, Oluwatosin A. Obisesan ^a^ and Samuel G. Awuah*^abcd^

aDepartment of Chemistry, University of Kentucky, Lexington, Kentucky 40506, United States

bCenter for Pharmaceutical Research and Innovation, Department of Pharmaceutical Sciences,

College of Pharmacy, University of Kentucky, Lexington, Kentucky 40536, USA

cMarkey Cancer Center, University of Kentucky, Lexington, Kentucky 40536, USA

^d^Center for Bioelectronics and Nanomedicine, University of Kentucky, Lexington KY, 40506, USA

[*Email](mailto:awuah@uky.edu): [awuah@uky.edu](mailto:awuah@uky.edu)

**SUPPORTING INFORMATION**

**Table of Contents**

Fig. S1: Effect of 2a and/or 2DG on cell cycle distribution in TNBC cell line MDA-MB-468 after 12 h of treatment. Dose (5mM 2DG and 1 μM 2a were used in both monotherapies and combination)

Fig. S2: Effect of 2a and/or 2DG on cell cycle distribution in TNBC cell line MDA-MB-468 after 24 h of treatment. Dose (5mM 2DG and 1 μM 2a were used in both monotherapies and combination)

Fig. S3: Effect of 2a and/or 2DG on cell cycle distribution in TNBC cell line MDA-MB-468 after 48 h of treatment. Dose (5mM 2DG and 1 μM 2a were used in both monotherapies and combination)

Fig. S4 Isobologram showing CI values for cotreatment of 9 mM 2DG and 2a (0.069, 0.21, 0.62, 1.85, 5.56, 16.7, 50 μM) in MDA-MB-468 cells

Fig. S5: S4 Isobologram showing CI values for cotreatment of 6 mM 2DG and 2a (0.069, 0.21, 0.62, 1.85, 5.56, 16.7, 50 μM) in MDA-MB-468 cells

Fig. S6: S4 Isobologram showing CI values for cotreatment of 3 mM 2DG and 2a (0.069, 0.21, 0.62, 1.85, 5.56, 16.7, 50 μM) in MDA-MB-468 cells

Fig. S7: Isobologram showing CI values for cotreatment of 9 mM 2DG and 2a (0.069, 0.21, 0.62, 1.85, 5.56, 16.7, 50 μM) in SUM159 cells

Fig. S8: Isobologram showing CI values for cotreatment of 6 mM 2DG and 2a (0.069, 0.21, 0.62, 1.85, 5.56, 16.7, 50 μM) in SUM159 cells

Fig. S9: Isobologram showing CI values for cotreatment of 3 mM 2DG and 2a (0.069, 0.21, 0.62, 1.85, 5.56, 16.7, 50 μM) in SUM159 cells

Table S1**.** Dose reduction index (DRI) of 9 mM 2DG in combination with 2a (0.069, 0.21, 0.62, 1.85, 5.56, 16.7, 50 μM) in MDA-MB-468 cells

Table S2**.** Dose reduction index (DRI) of 6 mM 2DG in combination with 2a (0.069, 0.21, 0.62, 1.85, 5.56, 16.7, 50 μM) in MDA-MB-468 cells

Table S3**.** Dose reduction index (DRI) of 3 mM 2DG in combination with 2a (0.069, 0.21, 0.62, 1.85, 5.56, 16.7, 50 μM) in MDA-MB-468 cells

Table S4**.** Dose reduction index (DRI) of 9 mM 2DG in combination with 2a (0.069, 0.21, 0.62, 1.85, 5.56, 16.7, 50 μM) in SUM159 cells

Table S5**.** Dose reduction index (DRI) of 6 mM 2DG in combination with 2a (0.069, 0.21, 0.62, 1.85, 5.56, 16.7, 50 μM) in SUM159 cells

Table S6**.** Dose reduction index (DRI) of 3 mM 2DG in combination with 2a (0.069, 0.21, 0.62, 1.85, 5.56, 16.7, 50 μM) in SUM159 cells

**Supplemental figures and tables**


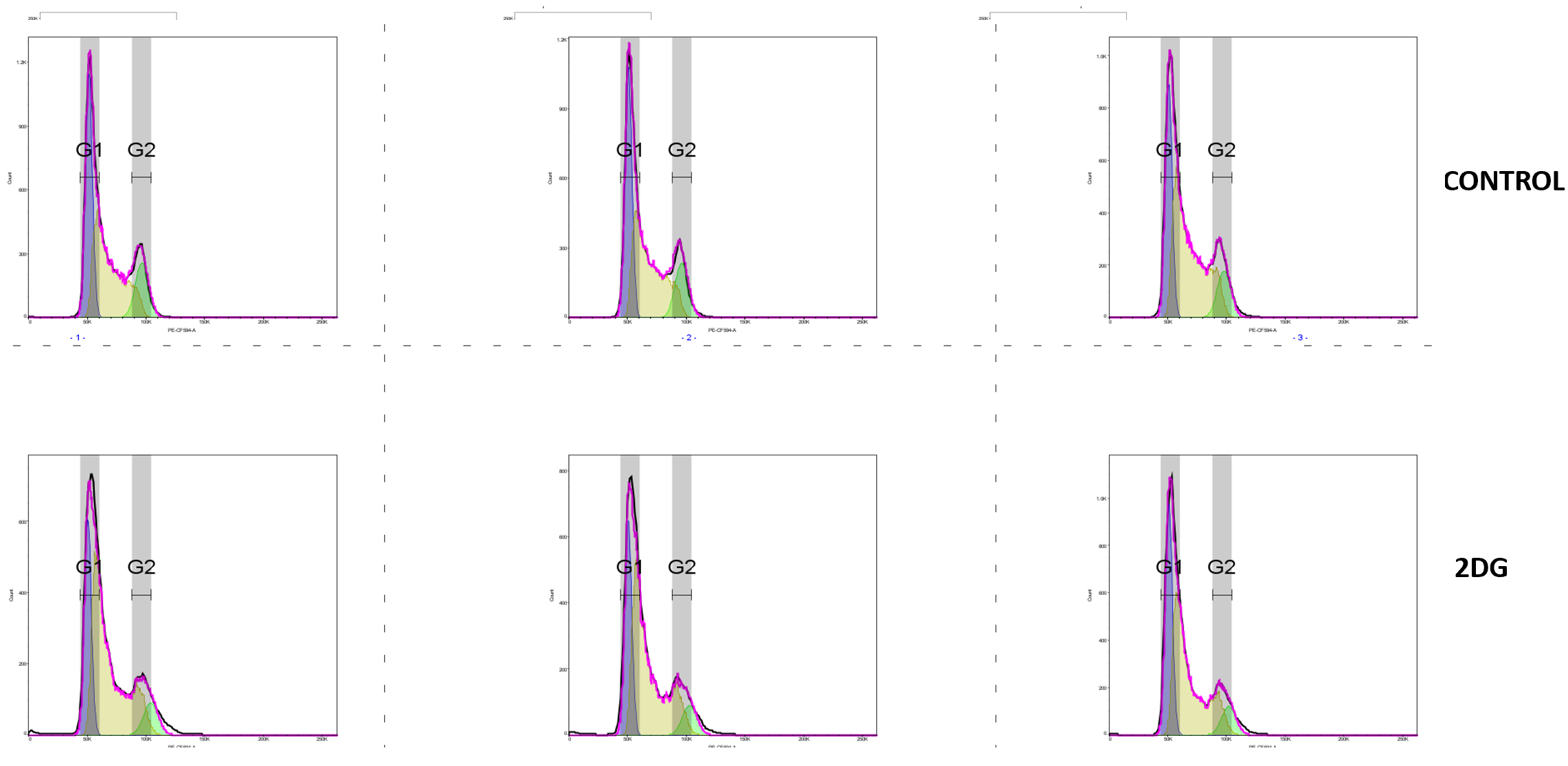


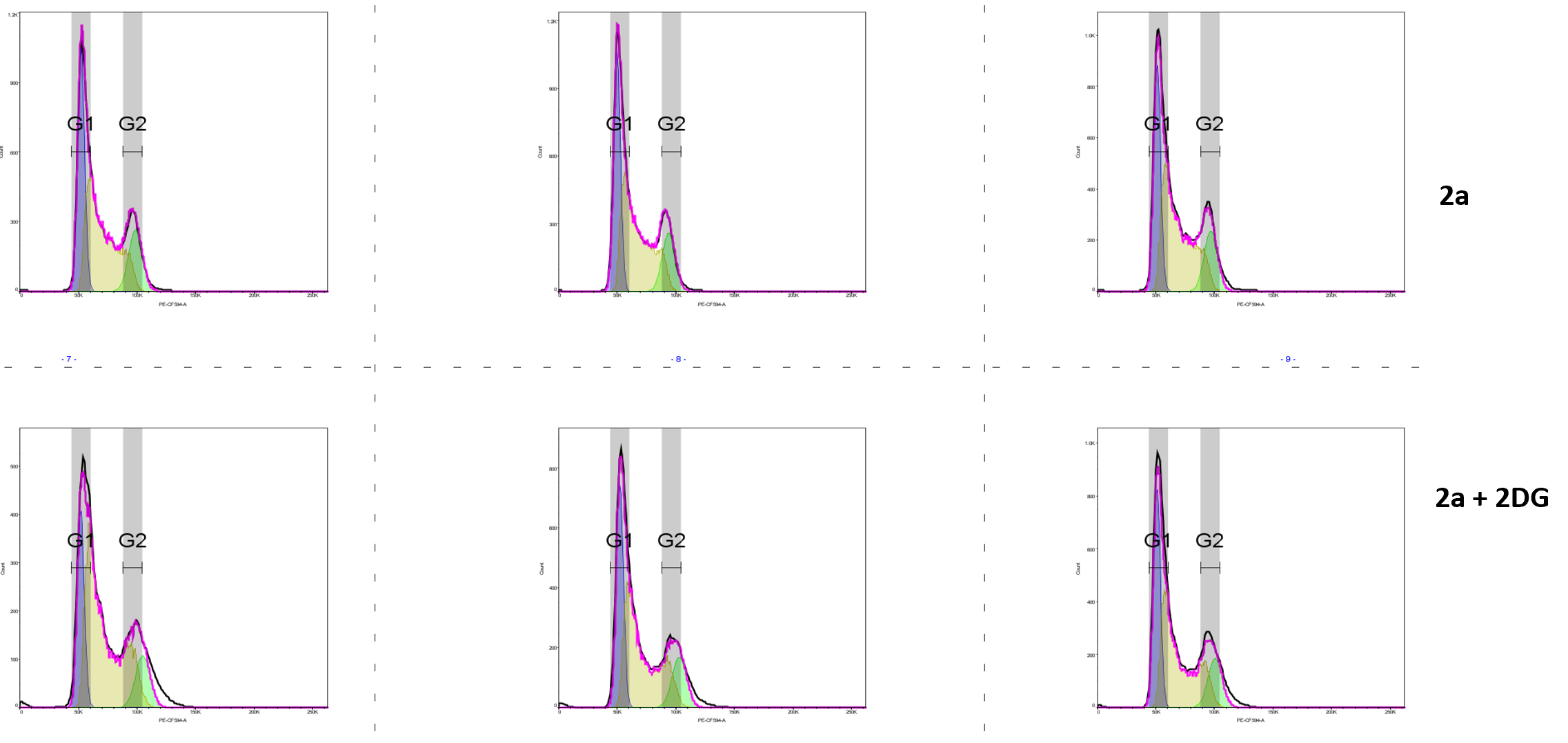


**Fig. S1**. Effect of 2a and/or 2DG on cell cycle distribution in TNBC cell line MDA-MB-468 after 12 h of treatment. Dosage (5mM 2DG and 1 μM 2a were used in both monotherapies and combinations.)


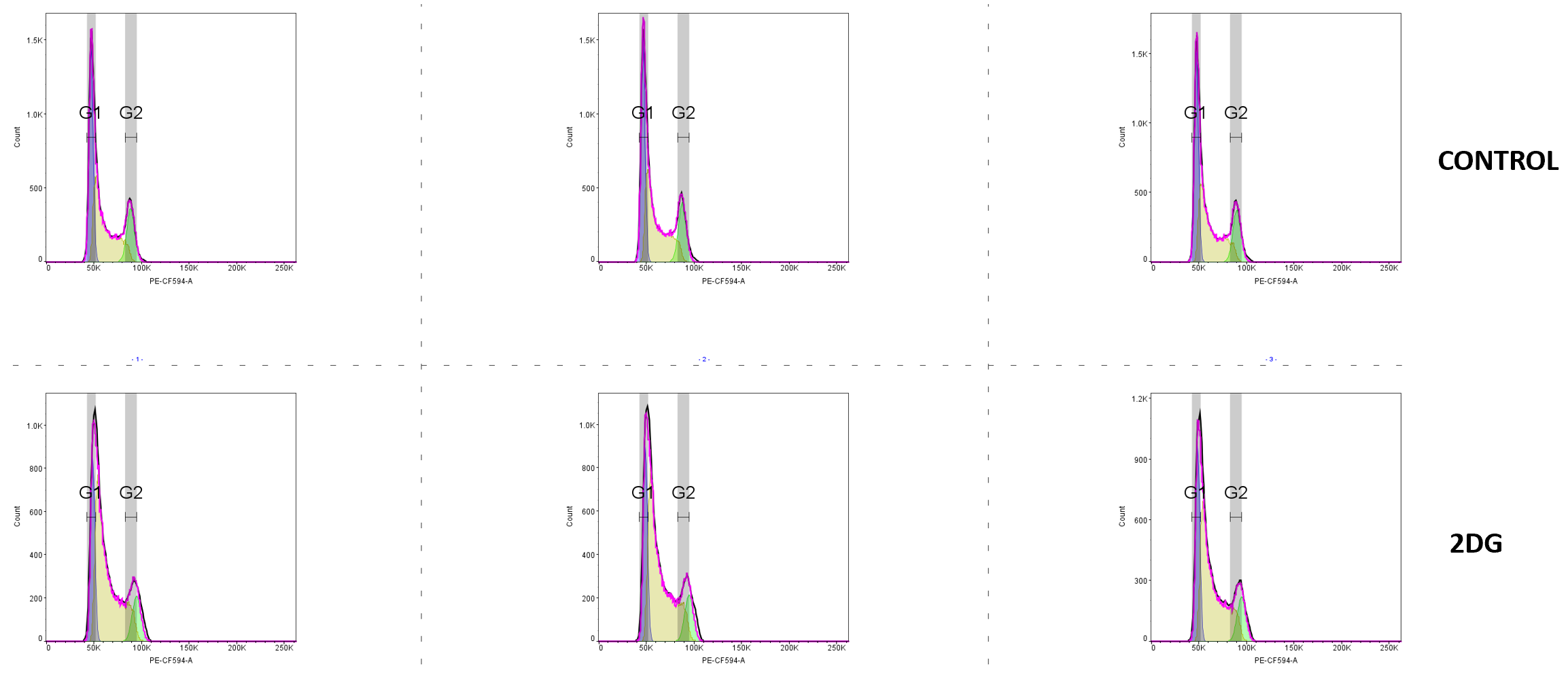


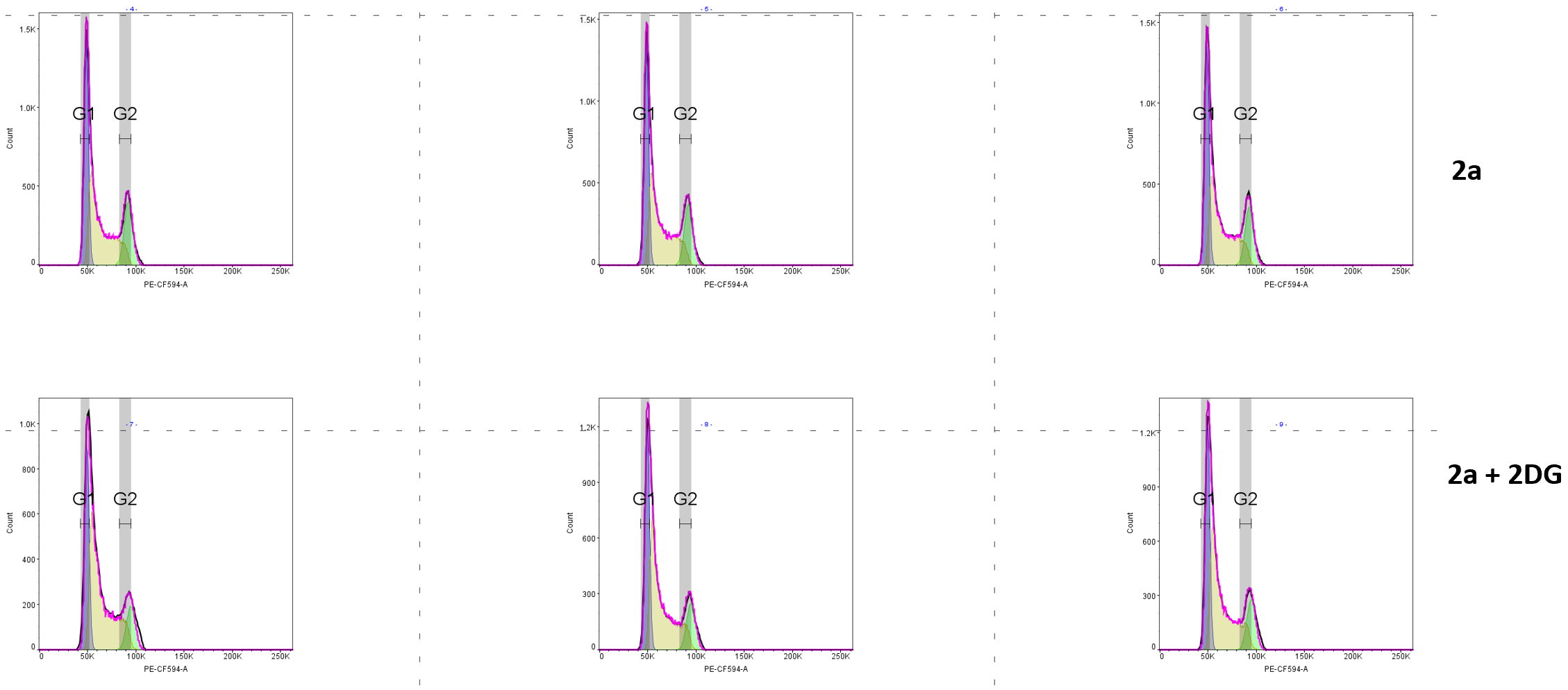


**Fig. S2**. Effect of 2a and/or 2DG on cell cycle distribution in TNBC cell line MDA-MB-468 after 24 h of treatment. Dosage (5mM 2DG and 1 μM 2a were used in both monotherapies and combinations.)


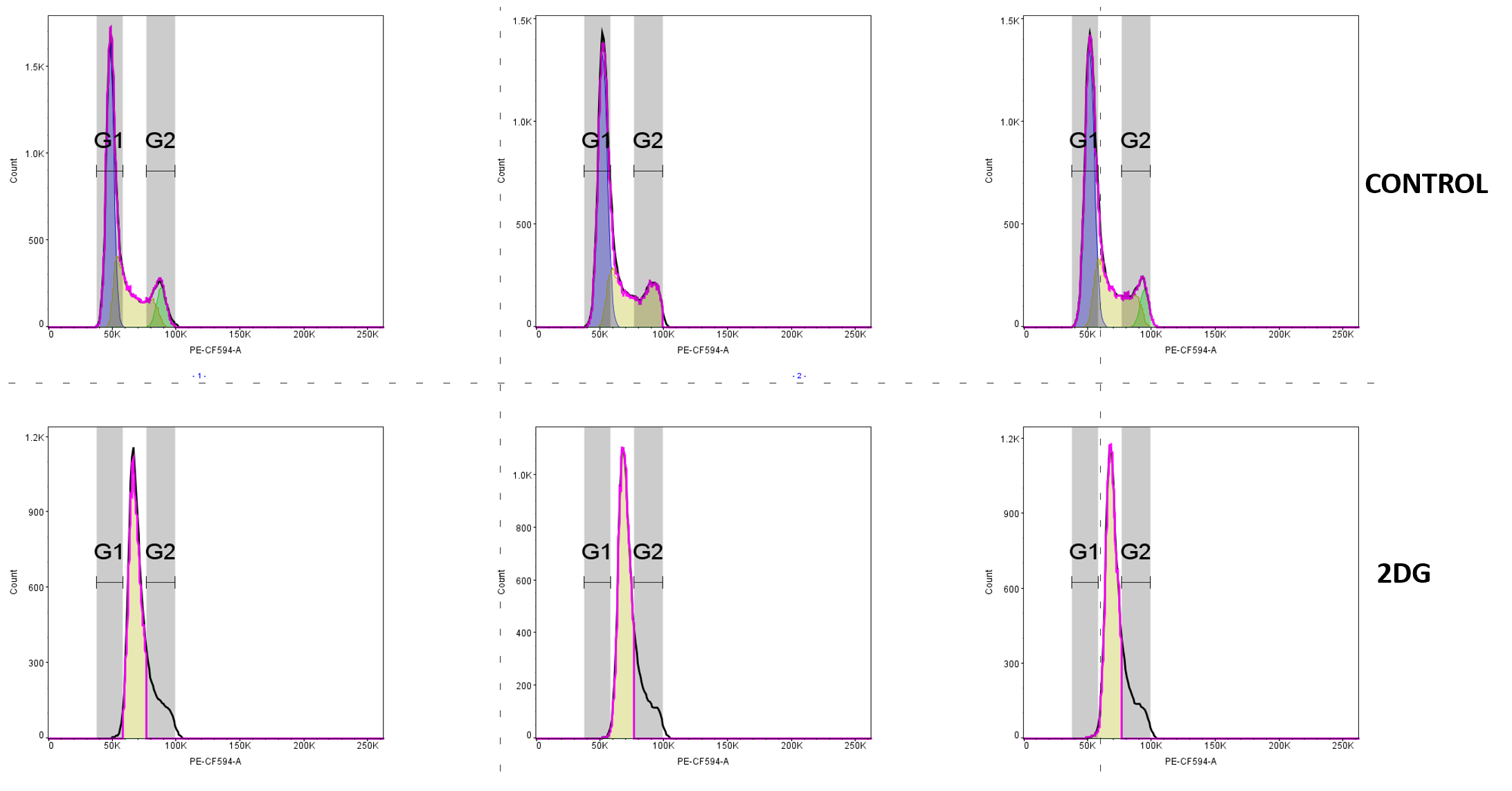


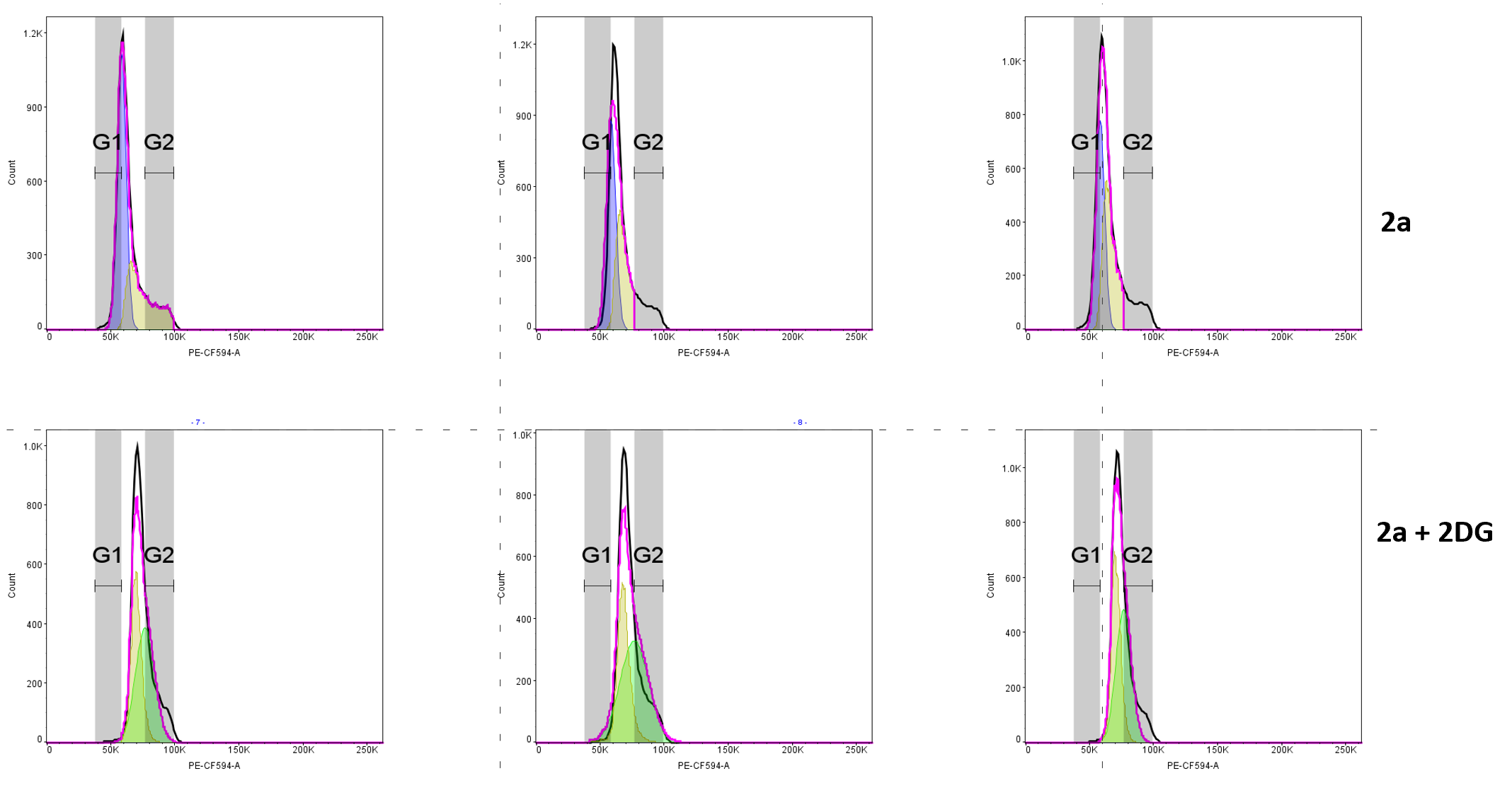


**Fig. S3.** Effect of 2a and/or 2DG on cell cycle distribution in TNBC cell line MDA-MB-468 after 48 h of treatment. Dosage (5mM 2DG and 1 μM 2a were used in both monotherapies and combinations.)


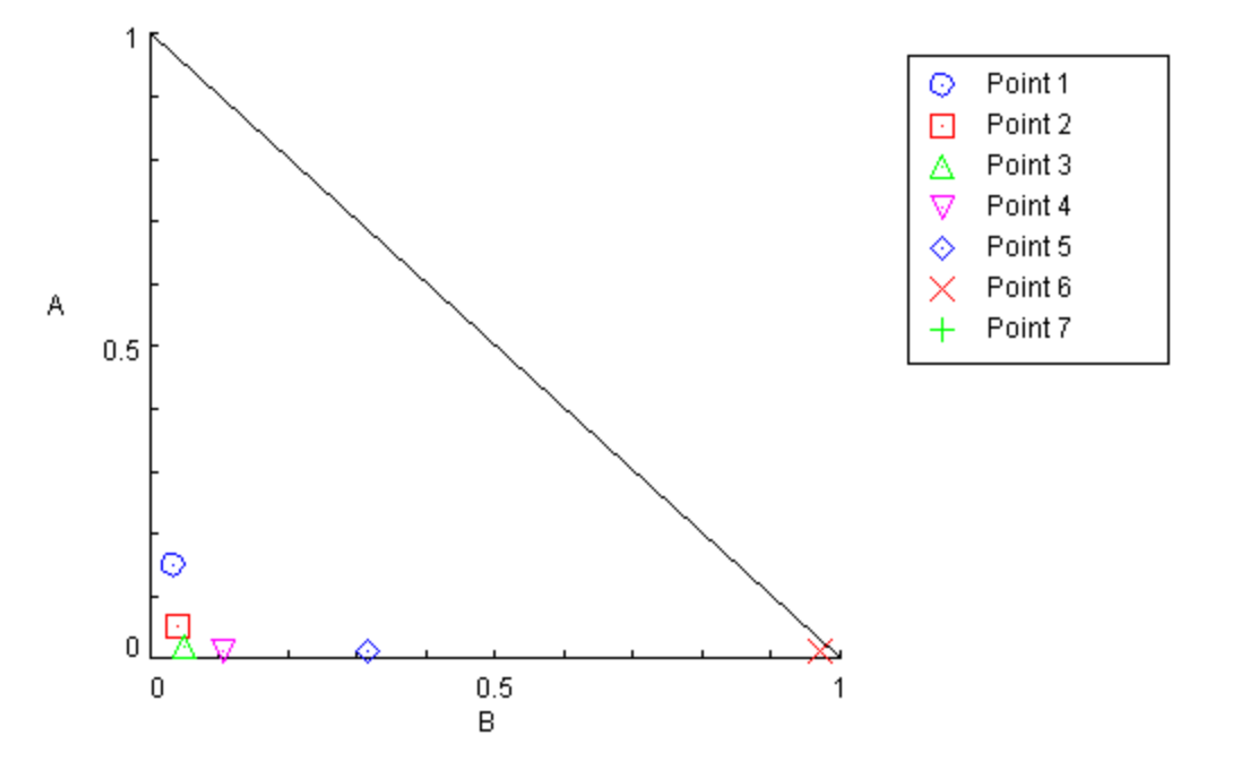


**Fig. S4.** Isobologram showing CI values for cotreatment of 9 mM 2DG and 2a (0.069, 0.21, 0.62, 1.85, 5.56, 16.7, 50 μM) in MDA-MB-468 cells


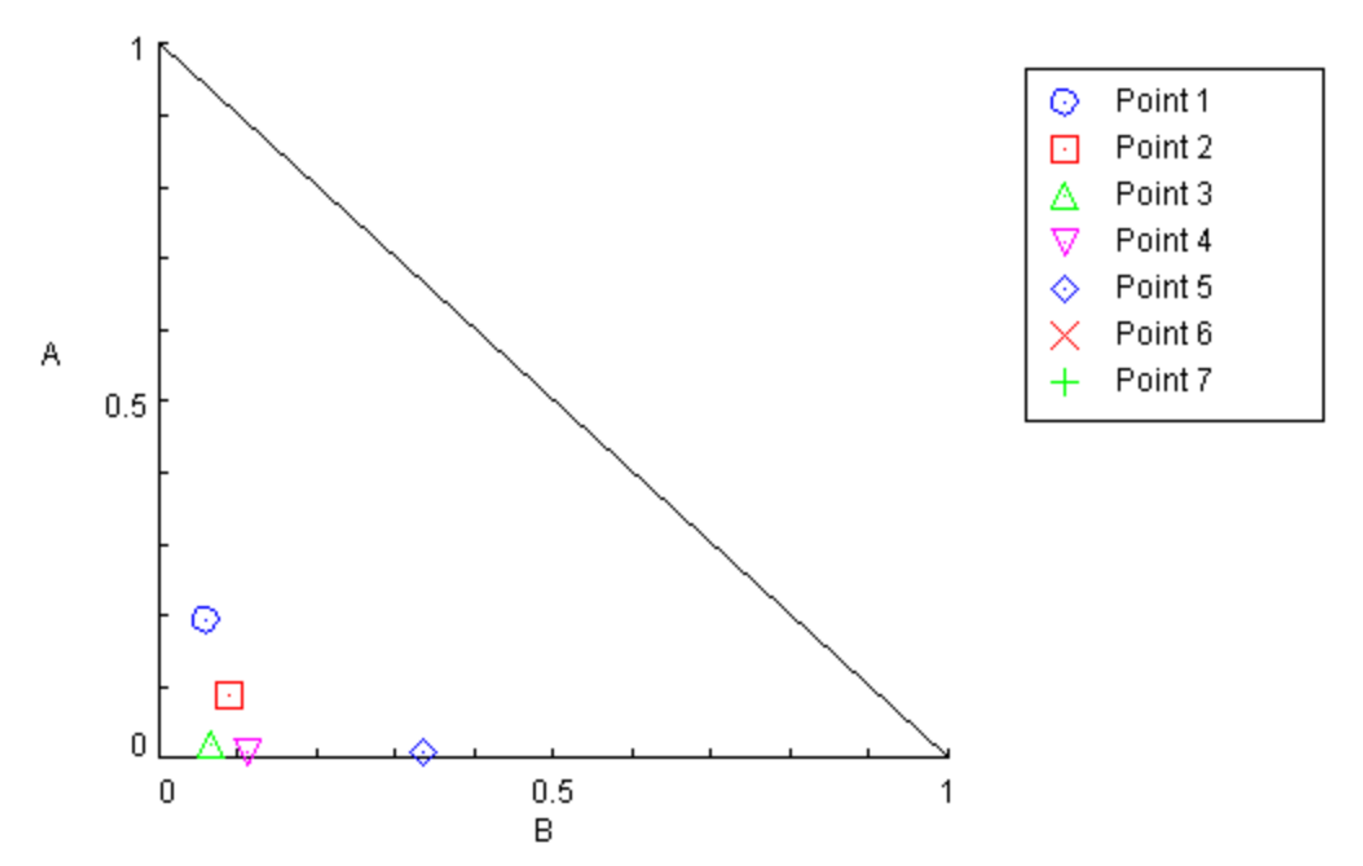


**Fig. S5.** Isobologram showing CI values for cotreatment of 6 mM 2DG and 2a (0.069, 0.21, 0.62, 1.85, 5.56, 16.7, 50 μM) in MDA-MB-468 cells.


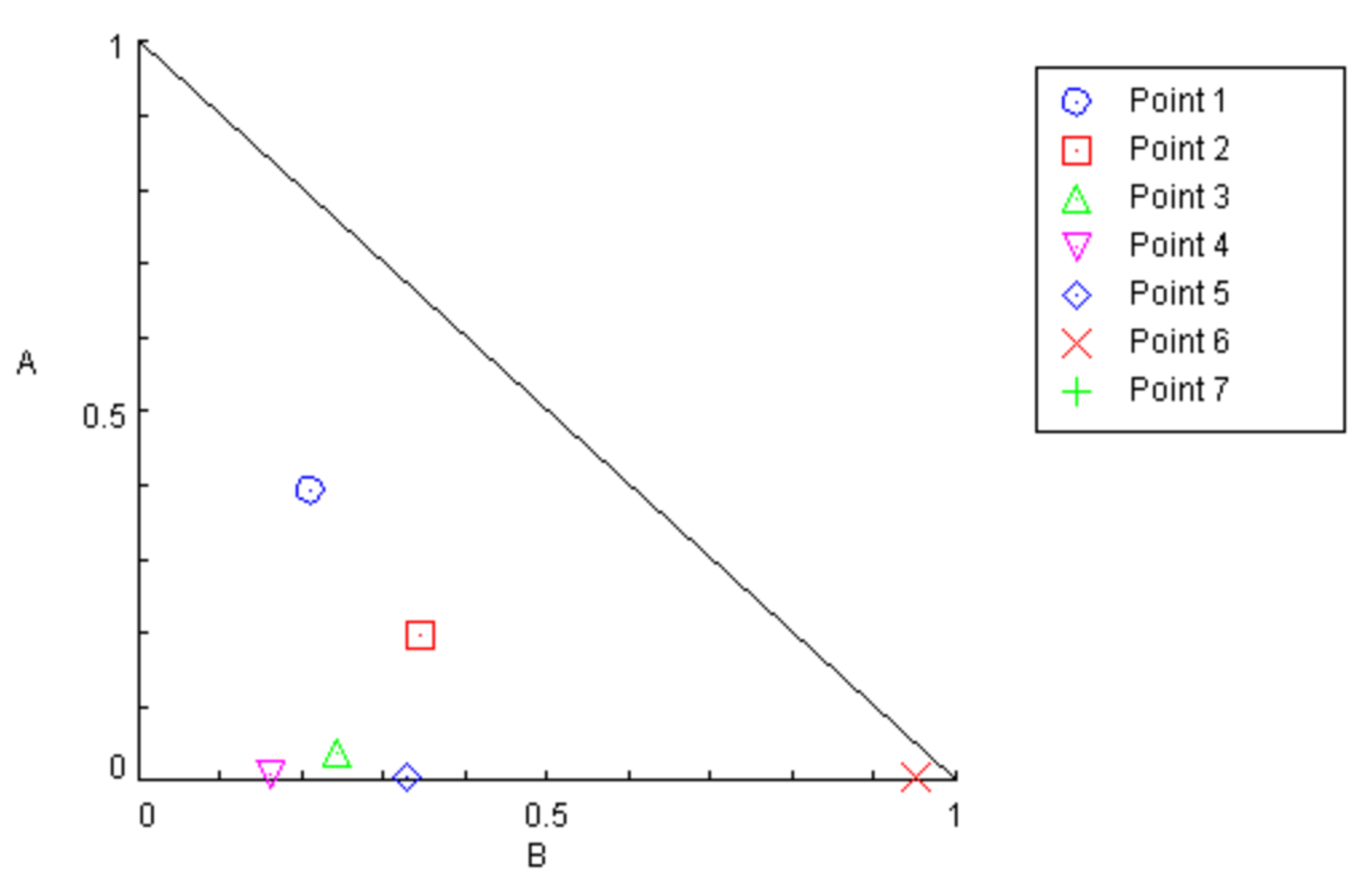


**Fig. S6.** Isobologram showing CI values for cotreatment of 3 mM 2DG and 2a (0.069, 0.21, 0.62, 1.85, 5.56, 16.7, 50 μM) in MDA-MB-468 cells.


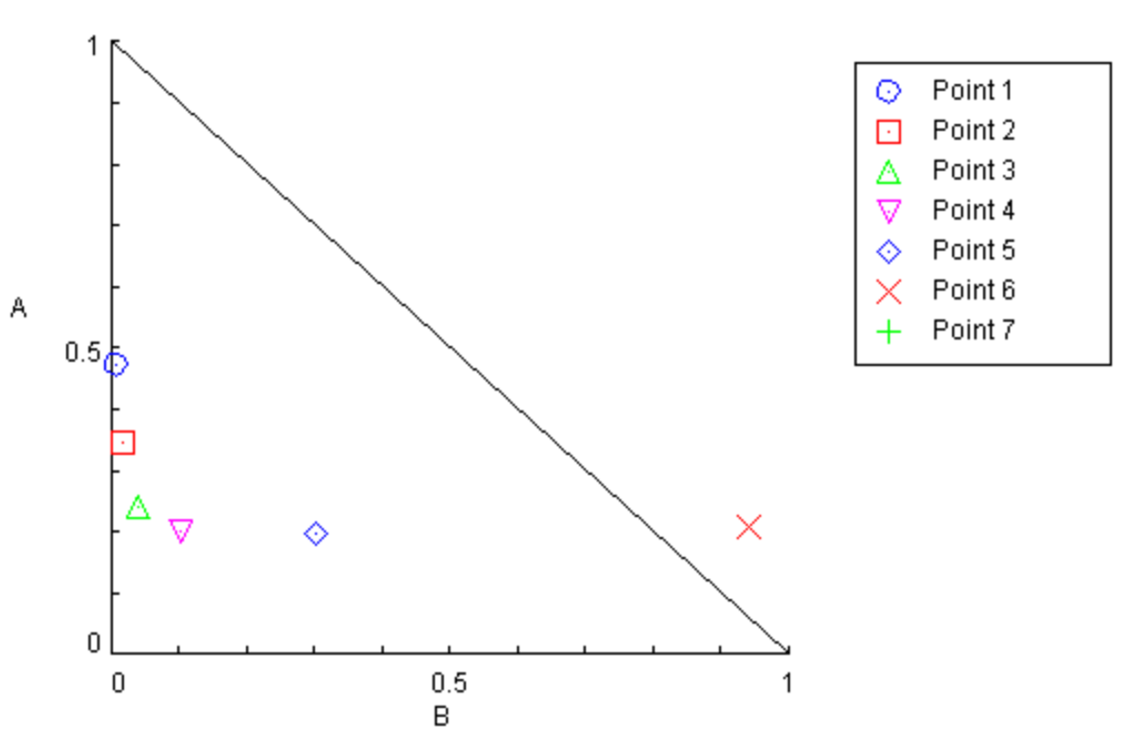


**Fig. S7.** Isobologram showing CI values for cotreatment of 9 mM 2DG and 2a (0.069, 0.21, 0.62, 1.85, 5.56, 16.7, 50 μM) in SUM159


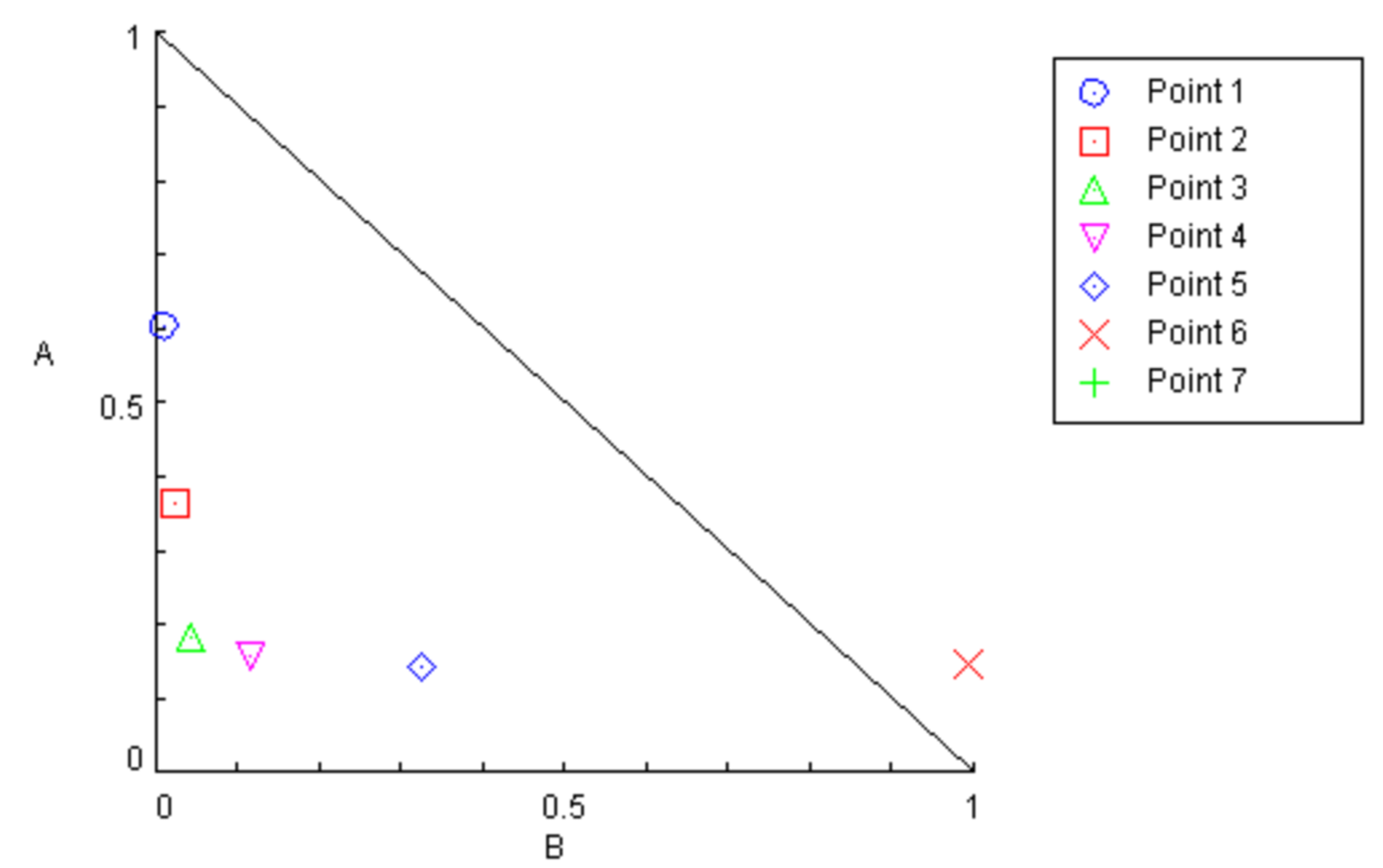


**Fig. S8.** Isobologram showing CI values for cotreatment of 6 mM 2DG and 2a (0.069, 0.21, 0.62, 1.85, 5.56, 16.7, 50 μM) in SUM159


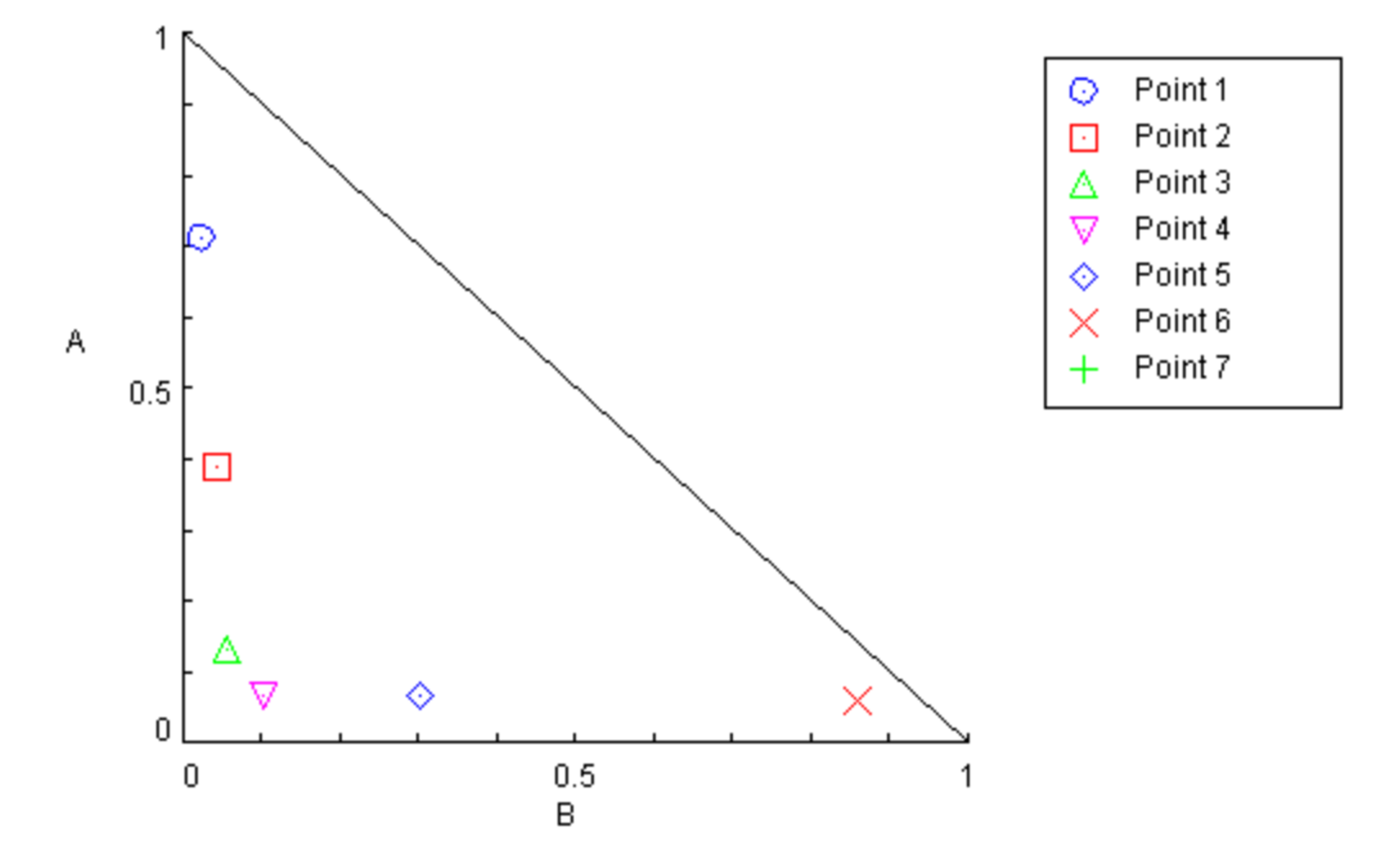


**Fig. S9.** Isobologram showing CI values for cotreatment of 3 mM 2DG and 2a (0.069, 0.21, 0.62, 1.85, 5.56, 16.7, 50 μM) in SUM159

**Table S1.** Dose reduction index (DRI) of 9 mM 2DG in combination with 2a (0.069, 0.21, 0.62, 1.85, 5.56, 16.7, 50 μM) in MDA-MB-468 cells


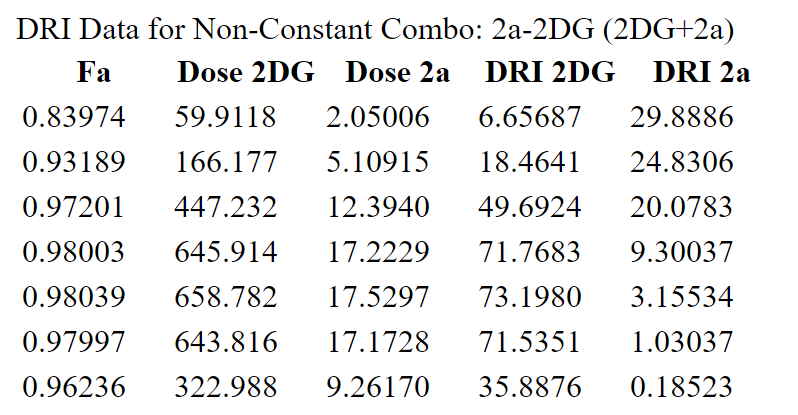


**Table S2.** Dose reduction index (DRI) of 6 mM 2DG in combination with 2a (0.069, 0.21, 0.62, 1.85, 5.56, 16.7, 50 μM) in MDA-MB-468 cells


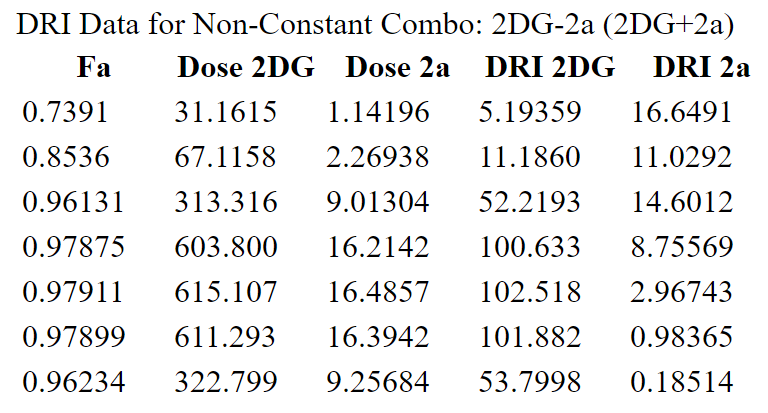


**Table S3.** Dose reduction index (DRI) of 3 mM 2DG in combination with 2a (0.069, 0.21, 0.62, 1.85, 5.56, 16.7, 50 μM) in MDA-MB-468 cells


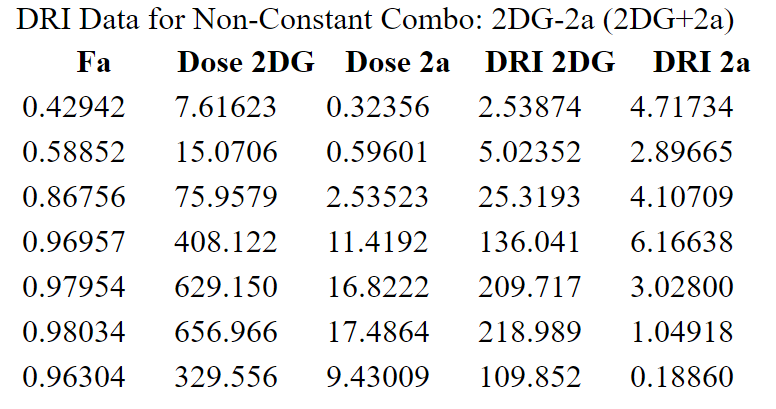


**Table S4.** Dose reduction index (DRI) of 9 mM 2DG in combination with 2a (0.069, 0.21, 0.62, 1.85, 5.56, 16.7, 50 μM) in SUM159 cells


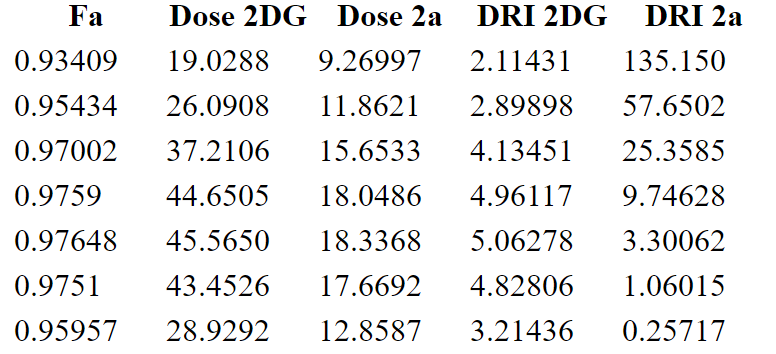


**Table S5.** Dose reduction index (DRI) of 6 mM 2DG in combination with 2a (0.069, 0.21, 0.62, 1.85, 5.56, 16.7, 50 μM) in SUM159 cells


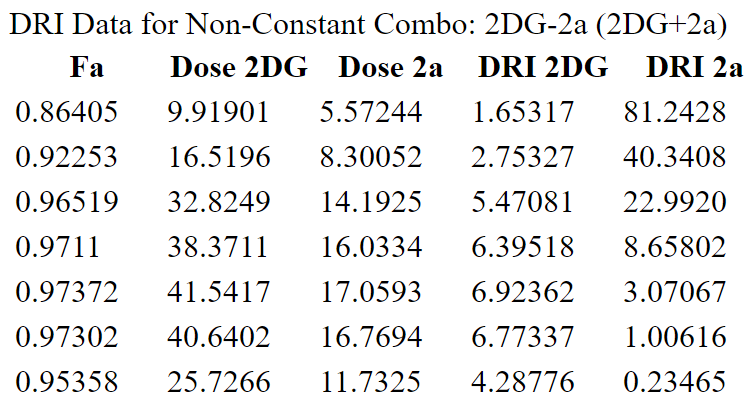


**Table S6.** Dose reduction index (DRI) of 3 mM 2DG in combination with 2a (0.069, 0.21, 0.62, 1.85, 5.56, 16.7, 50 μM) in SUM159 cells


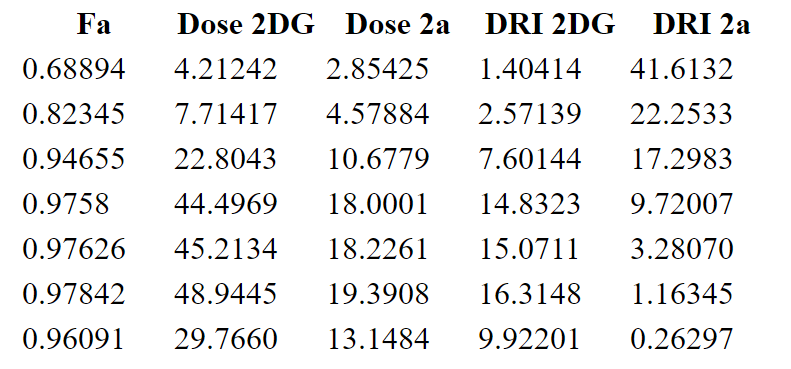

Supplement: Supporting information [file mmc1.docx]
